# Compensatory respiration supports survival during extended heat stress in *Microcystis aeruginosa*

**DOI:** 10.1126/sciadv.adz4338

**Published:** 2026-06-03

**Authors:** Oded Liran, Eli Shemesh, Dan Tchernov, Kevin J. Kunstman, Stefan J. Green, Surabhi Naik, Hagit Zer, Nir Keren, Assaf Sukenik, Reham Kh. Khalil

**Affiliations:** ^1^ Yigal Alon Kinneret Limnological Institute, Israel Oceanographic and Limnological Research, Migdal 149500, Israel.; ^2^ Leon Charney School of Marine Sciences, University of Haifa, Haifa 345000, Israel.; ^3^ Genomics and Microbiome Core Facility, Rush University, Chicago, IL 60612, USA.; ^4^ Rush Research Bioinformatics Core, Rush University, Chicago, IL 60612, USA.; ^5^ Alexander Silverman Institute of Life Sciences, Hebrew University of Jerusalem, Givat Ram, Jerusalem 9190401, Israel.; ^6^ Tel-Hai Academic College, Upper Galilee 1220800, Israel.

## Abstract

Phototrophic organisms must balance energy between photosynthesis and respiration to survive. In cyanobacteria, this coordination is especially critical as both processes share the same membrane and electron transport complexes. Although electron exchange between the two pathways is known, photosynthesis is often presumed to dominate under many conditions. Here, under an extended heat-stress condition, our data indicate that respiration can compensate for reduced photosynthetic activity. We compared two *Microcystis aeruginosa* strains (PCC7806 and Lake Kinneret isolated strain C-1004). PCC7806 maintained viability while increasing dark respiration and sustaining a larger, active plastocyanin pool and turnover. C-1004 maintained higher photosystem II activity but showed reduced respiration and reduced biomass. Overall, the results suggest that respiratory compensation, rather than residual photochemical throughput, aligns with survival under this condition. Such differences could contribute to seasonal shifts in strain dominance and point to respiration as an important survival mechanism in cyanobacteria during heat stress.

## Teaser

Dark respiration rises during heat stress in a resilient *Microcystis* strain, pointing to a backup when photosynthesis fails.

## INTRODUCTION

*Microcystis aeruginosa* is one of the most common bloom-forming cyanobacteria in freshwater ecosystems worldwide (*1*). These organisms can adapt to wide range of environmental conditions. These include a range of light intensities (*2*) and wide temperature tolerance from 12° to 30°C in situ and to 35°C in vitro (*3*, *4*). These characteristics, together with a gas vesicle–related buoyancy mechanism, turn *M. aeruginosa* into an ecologically dominant microorganism (*5*). *Microcystis* blooms can result in serious environmental events: release of toxins into water bodies (*6*–*8*), blocking drinking water supply systems, and producing unpleasant odors that deteriorate water quality. In recent years, the frequency and intensity of *Microcystis* blooms have increased, accompanied by their expansion into previously unidentified geographic regions and ecological niches (*8*). These trends are largely driven by gradual environmental changes associated with climate change, rising CO_2_ levels, and eutrophication (*9*). As freshwater ecosystems experience more frequent and extended heatwaves, the survival strategies of dominant cyanobacteria such as *M. aeruginosa* have come under renewed scrutiny.

**Fig. 1.**
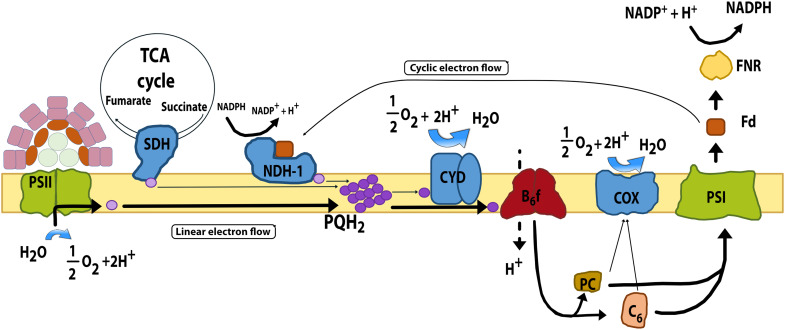
Shared thylakoid electron transport chain links photosynthesis and respiration in cyanobacteria. Schematic diagram of the main photosynthetic and respiratory complexes embedded in the cyanobacterial thylakoid membrane. Electrons can enter the chain from photosystem II (PSII), termed linear electron flow, or from respiratory dehydrogenases and be transferred either to photosystem I (PSI) or to terminal oxidases. Electrons can be transferred back to the plastoquinone (PQ) pool through reduced ferredoxin (Fd) transferring electrons to back to NDH-1, termed cyclic electron flow. As a result, photosynthesis and respiration draw on the same electron carriers. This arrangement creates key junctions at the PQ pool and at the plastocyanin (PC)/cytochrome c_6_ step, where the shared redox state of the electron carriers is determined by the combined activity of photosynthesis and respiration. Photosynthetic complexes PSII and PSI are shown in light green. The membrane-bound electron carrier PQ is shown in pale purple, and its reduced form plastoquinol (PQH_2_) represents the PQ pool (dark purple). The cytochrome b_6_f complex is colored in Bordeaux. The soluble electron carriers PC and cytochrome c_6_ are colored in baize and light brown, respectively. Soluble Fd and Fd:NADP^+^ reductase (FNR) are shown in brown and yellow, respectively. Respiratory membrane complexes are indicated in blue: type I NAD(P)H dehydrogenase (NDH-1), succinate dehydrogenase (SDH), and the terminal oxidases cytochrome bd (CYD) and cytochrome c oxidase (COX).

Unlike chloroplasts in algae and plants, cyanobacterial thylakoid membranes integrate multiple energy metabolism pathways (*10*–*12*). They house both the photosynthetic electron transport chain and components of the respiratory electron transport chain (Fig. 1). In cyanobacteria, photosystem II (PSII) is excited via the phycobilisome light-harvesting complex (LHC). It then reduces the plastoquinone (PQ) pool. PQ can also be reduced by succinate dehydrogenase (SDH) from the tricarboxylic acid (TCA) cycle and by the type I reduced form of nicotinamide adenine dinucleotide phosphate (NADP^+^) [NAD(P)H] dehydrogenase (NDH-1) from the central metabolism. Reduced PQ (PQH_2_) either transfers electrons to the cytochrome b_6_f complex or to the bd quinol oxidase (CYD). The b_6_f complex reduces the soluble carriers plastocyanin (PC) and cytochrome c_6_, which then deliver electrons either to photosystem I (PSI) (*13*) or to the cytochrome c oxidase (COX). Electrons leaving PSI reduce the soluble electron carrier ferredoxin (Fd), which is used by Fd:NADP^+^ reductase (FNR) to generate NADPH for metabolism. Alternatively, electrons can be routed back to the PQ pool via connection of Fd to NDH-1 complexes [cyclic electron flow; (*14*)] or directly from consumption of NADPH by this complex (*15*). Certain cyanobacteria have additional respiratory activity at the cytoplasmic membrane [e.g., the alternative respiratory terminal oxidase (ARTO); (*16*)]. Its presence is species dependent and often stress induced.

Previous work shows that, under steady-state growth, cyanobacterial dark respiration reaches up to approximately one-fifth of gross photosynthetic oxygen evolution (*17*). It therefore contributes only a minor fraction of total oxygen flux and varies systematically with growth light and physiological state. Moreover, respiration can be light enhanced during or immediately after illumination (*18*). In diel cycles, phosphoketolase and 2-dehydro-3-deoxy-phosphogluconate aldolase pathways further modulate photosynthetic carbon yield (*19*). Respiration has also been shown to contribute to electron flow during illumination. For example, mutants lacking PSI were able to sustain only marginal growth at very low light intensities (*20*). This indicates that respiratory complexes can donate electrons to the transport chain, albeit at electron transport rates much lower than those driven by PSII-based photochemistry (*13*). Last, under nutrient-limiting conditions, respiratory oxygen uptake has been reported to reach up to approximately half of the photosynthetic oxygen evolution rate (*21*).

In cyanobacteria, respiration has long been recognized as an auxiliary electron valve. Particularly under high-light stress, terminal oxidases reoxidize the PQ pool to prevent overreduction (*18*, *22*–*24*). Respiration is also strongly temperature dependent, increasing with rising temperatures in a variety of phytoplankton species (*25*). Yet despite this, the role of respiration during heat stress periods in cyanobacteria has received little direct attention. Most studies that focused on short period of heat-shock responses considered respiration only indirectly, at transcript level in general, or to explain central metabolism response (*26*, *27*). This gap highlights the need for physiological studies that examine how the photosynthesis-respiration coupling contributes to thermal resilience in cyanobacteria.

In the past decade, big attention has been devoted to understanding how cyanobacteria acclimate to heat shock. This includes acclimation to various elevated temperatures and the underlying mechanisms that enable them to withstand the shock (*28*, *29*). Most research has focused on heat shock proteins (HSPs) and their rapid induction following the onset of heat stress (*26*, *30*). Given the high sensitivity of PSII to temperature, further investigations in this area have uncovered several important findings: (i) an increase in PsbO subunit, which stabilizes the manganese cluster at the PSII site (*31*), (ii) membrane fluidity influence that affects overall PSII stability (*32*), and (iii) increase in D1 subunit transcripts even at moderate temperature elevations (*27*). Additionally, at the genome level, HSP regulation in cyanobacteria was found to be mediated by cis-elements that recruit specialized regulators, namely, the Hik34-Rre1 regulator system (*33*). Hik34-Rre1 system responds to a short consensus motif (GTnCGG) upstream to induced genes during the shock, reported as the minimal Rre1 binding sequence (*33*).

Unlike many *Microcystis* populations around the world that bloom during warm seasons, *Microcystis* in Lake Kinneret form blooms during winter months at temperatures of ~15° to 19°C (*34*). Hydroacoustic surveys have shown that dense *Microcystis* layers in Lake Kinneret can develop when mixed-layer temperatures are around 16° to 18°C at water surface (*35*). This indicates that, in this system, *Microcystis* attains high biomass at lower temperatures than are usually reported for *Microcystis* blooms in other lakes. As temperatures rise toward spring and early summer, these winter *Microcystis* blooms typically decline. They are followed by peaks of the dinoflagellate *Peridinium gatunense* (*36*), which now appears more irregularly but often becomes dominant when *Microcystis* declines (*37*). This unique seasonal pattern raises an important question: Why does the isolated Lake Kinneret strain (C-1004) bloom under cooler conditions, and can it maintain a competitive advantage as temperatures increase?

In controlled experiments, it is customary to test the limits of photosynthetic organisms to temperature by exposing them to abrupt, supraecological heat shocks. Such treatments, typically involve rapid temperature shifts of 20° to 30°C at once. These are widely used to probe the limits of the protection pathways: stability of PSII, membrane integrity, and the capacity of molecular chaperones (*38*–*43*). Although these experimental conditions do not replicate the gradual diel or seasonal warming in natural environments, they serve as boundary tests, revealing bottlenecks and survival strategies that remain hidden under milder changes. Recent extreme warming events in freshwater systems, including lakes approaching or exceeding 40°C during severe heat waves, further emphasize the relevance of examining responses near the upper thermal limit (*44*).

Building on this approach, we investigated the physiological responses of the *M. aeruginosa* strain C-1004, isolated in the past from Lake Kinneret, to the well-characterized reference *M. aeruginosa* strain PCC7806. On the basis of its natural behavior in situ, we hypothesized that C-1004 would have a reduced ability to tolerate extended thermal stress relative to PCC7806. To test this, we subjected both strains to a step shift from growth temperature at 20° to 40°C for 48 hours. This temperature is ultimately lethal under extended exposure but can be sustained transiently for several days. While such transition conditions are unlikely to occur in Lake Kinneret, they allow us to uncover contrasting physiological survival strategies. Such strategies may influence the ecological success of different *Microcystis* strains under future warming scenarios.

## RESULTS

**Fig. 2.**
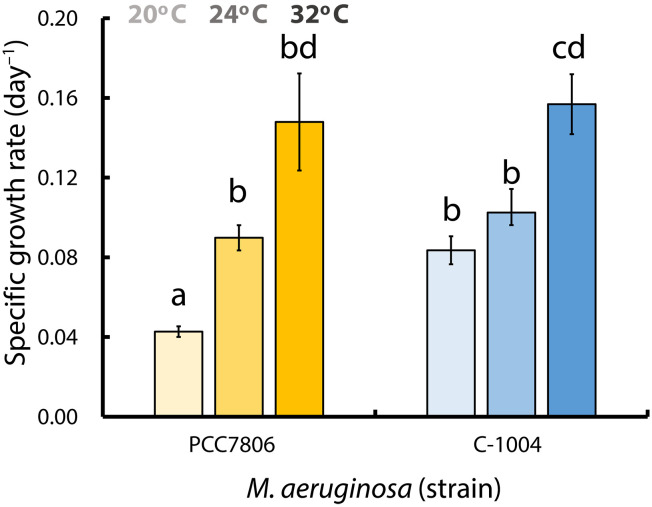
Cold temperature gives C-1004 a growth advantage, while growth rates converge at higher temperatures. Specific growth rates (day^−1^) of *M. aeruginosa* strains PCC7806 (yellow) and C-1004 (blue) grown at 20°, 24°, and 32°C. The *y* axis shows specific growth rate in units of (day^−1^); the *x* axis shows the two strains, with bar groups representing the three temperatures. Darker bar shades indicate higher temperatures. Bars show means ± SEM (*n* = 4 biological replicates). Differences among all strain-temperature combinations were tested using a one-way analysis of variance (ANOVA) followed by post hoc independent-samples two-tailed *t* tests (Welch’s *t* test when variances were unequal). Bars that share the same letter do not differ significantly. Bars with different letters differ significantly (*P* < 0.05).

**Fig. 3.**
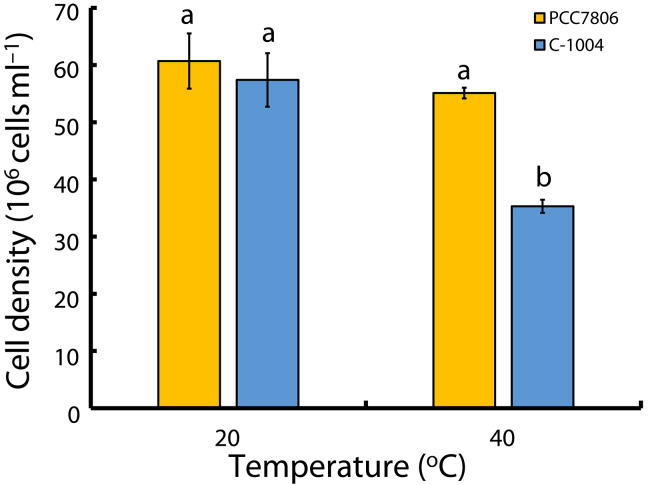
Forty-eight–hour exposure of 40°C reduces cell density in C-1004, but not in PCC7806. Cell densities of *M. aeruginosa* strains PCC7806 (yellow) and C-1004 (blue) before and after extended heat stress at 20° or 40°C. The *y* axis shows cell density (10^6^ cells ml^−1^); the *x* axis shows the incubation temperature. Cultures were inoculated at similar initial cell densities at 20°C and then shifted to 40°C for 48 hours. Bars show means ± SEM (*n* = 3 biological replicates). Within-strain effects of temperature were tested using repeated-measures ANOVA with Greenhouse-Geisser correction when sphericity was violated, followed by paired two-tailed *t* tests. Between-strain differences at each temperature were tested by one-way ANOVA followed by independent-samples two-tailed *t* tests (Welch’s *t* test when variances were unequal). Letters above the bars indicate groups that differ significantly across all comparisons (*P* < 0.05).

Two strains of *M. aeruginosa* were acclimated to three temperatures regimes: 20°, 24°, or 32°C. They were maintained in continuous serial batch culture for at least 10 growth cycles. Each cycle consisted of growth under controlled exponential conditions, dilution when culture reached stationary phase, and reinoculation. At 20°C, this acclimation period lasted ~10 months, whereas, at higher temperatures, it was achieved more quickly (Fig. 2). The local strain, C-1004, exhibited a growth rate twice as high as the widely studied PCC7806 at 20°C (*P* = 0.0016). When we attempted to grow the cultures at 40°C, neither strain persisted for long: PCC7806 maintained its biomass for ~3 to 4 days after transfer, whereas C-1004 declined more rapidly, and, by day, 7 both cultures had ceased growth, were strongly bleached, and exhibited quantum yields of PSII that had dropped to zero. This prompted us to ask how the two strains, PCC7806 and C-1004, would cope with a temperature step shift, a direct transfer from 20° to 40°C. To assess survival under extended heat stress, we quantified cell density before the heat shock at 20°C and after 48 hours at 40°C (Fig. 3). Both strains began at comparable densities. After 48 hours, PCC7806 remained near its initial density, whereas C-1004 declined in at least one-third relative to the density at the beginning of the experiment (*P* = 0.0397), indicating reduced survival.

**Fig. 4.**
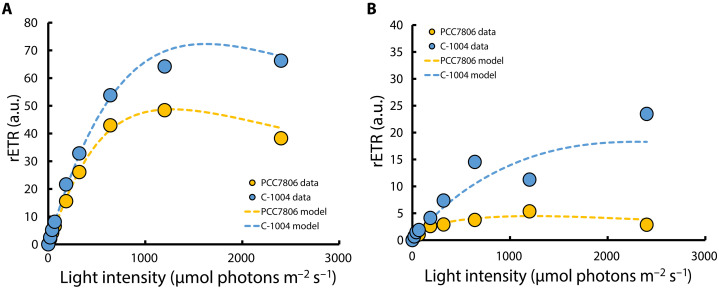
PSII activity is largely lost in PCC7806 but partly retained in C-1004 after extended heat stress. Rapid light curves (RLCs) of *M. aeruginosa* strains PCC7806 (yellow) and C-1004 (blue) measured at 20°C (**A**) and after 48 hours at 40°C (**B**). The *y* axis shows the apparent PSII photochemical performance (relative units; derived from RLC measurements), and the *x* axis shows actinic irradiance in photon photosynthetic flux density (μmol photons m^−2^ s^−1^). Data points represent the mean of three biological replicates; dashed lines are model fits to the measured points (*79*). For improved resolution, the *y* axis scale in (B) is half that of (A).

If the C-1004 strain experienced difficulty acclimating to high temperature, then the first photosynthetic complex likely to be impaired would be PSII, which is generally the most heat-sensitive component of the photosynthetic electron transport chain. Under thermal stress, PSII is particularly heat sensitive, with disruption of the oxygen-evolving complex and impaired D1 turnover contributing to rapid loss of activity (*32*, *45*). To test whether PSII function was affected under these conditions, we measured PSII performance across a range of light intensities using rapid light curves (RLCs) before the temperature shift and after 48 hours at 40°C (Fig. 4 and table S1). At 20°C, both strains exhibited typical RLC profiles (Fig. 4A). C-1004 reached a maximal relative electron transport rate (rETR) ~1.5 times higher than that of PCC7806 at 1200 μmol photons m^−2^ s^−1^. This reflects a higher PSII electron transport capacity in C-1004 under saturating light at this temperature. At the culture light intensity (35 μmol photons m^−2^ s^−1^), there was no measurable difference between the strains, consistent with the fact that RLC test probes short-term PSII responses rather than steady-state growth performance. C-1004 showed a plateau in its response, maintaining stable PSII activity even at 2400 μmol photons m^−2^ s^−1^ (Fig. 4, compare the two strains at the highest light intensity). After extended heat stress, PSII activity decreased in both strains, consistent with the expected thermal sensitivity of PSII (Fig. 4B). PCC7806 activity dropped sharply to just 10% of its prestress maximum, whereas C-1004 retained roughly one-third of its original PSII activity.

**Fig. 5.**
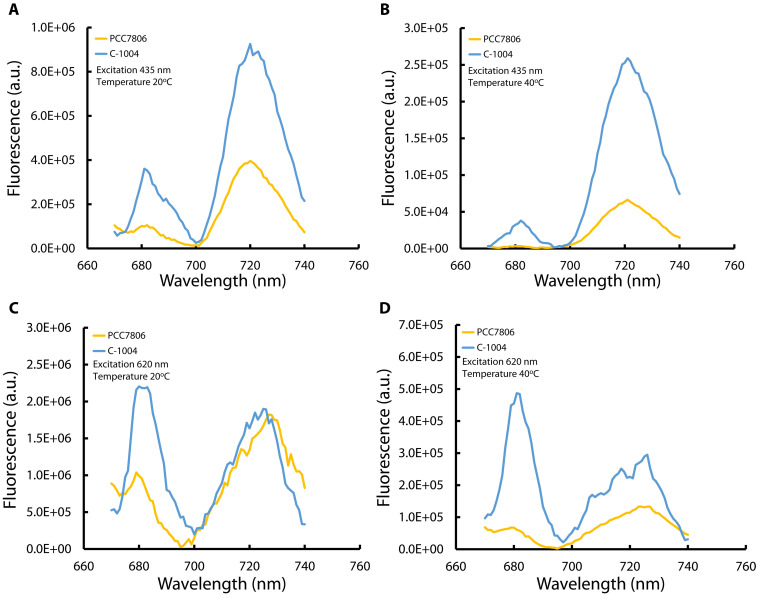
Extended heat stress induces contrasting state transitions and LHC complexes redistribution in PCC7806 and C-1004 strains. Fluorescence emission spectra (77 K) of *M. aeruginosa* strains PCC7806 (yellow) and C-1004 (blue) measured at 20°C (**A** and **C**) and after 48 hours at 40°C (**B** and **D**). [(A) and (B)] Emission spectra following excitation at 435 nm, primarily reflecting chlorophyll a fluorescence associated with PSII and PSI. [(C) and (D)] Emission spectra following excitation at 620 nm, highlighting the distribution of excitation energy absorbed by the light-harvesting phycobilisome antenna, between PSII and PSI. In all panels, curves represent the mean of three biological replicates at 20°C and two biological replicates after 48 hours at 40°C.

The decrease in PSII electron transport rate suggests that the photosynthetic apparatus may have reorganized under heat stress. Such reorganization could involve changes in the balance between PSII and PSI or redistribution of excitation energy between the two photosystems. These adjustments could reduce electron generation at a malfunctioning PSII reaction center, which is especially sensitive to elevated temperatures (*32*). Because C-1004 exhibited an approximately two-thirds reduction in PSII activity under heat stress (Fig. 4 and table S1), we examined the relative PSII and PSI contribution and possible energy redistribution using 77-K fluorescence analysis (Fig. 5). In cyanobacteria, PSI-associated fluorescence emission at 77 K is often more pronounced than PSII-associated emission, consistent with a relatively high PSI:PSII ratio and the fact that a substantial fraction of cellular chlorophyll a is associated with PSI (*46*, *47*). This pattern was also observed under control conditions in our strains (Fig. 5A). Based on the relative heights of the PSI- and PSII-associated bands, the PSI:PSII fluorescence contribution was ~2:1 in C-1004 and ~4:1 in PCC7806 (Fig. 5A). In parallel, the PSII-associated fluorescence peak of C-1004 was about fourfold higher than that of PCC7806, consistent with the higher PSII electron transport rates measured in C-1004. After 48 hours at 40°C, total fluorescence decreased to approximately one-third of the control level in both strains (Fig. 5B). Under these conditions, C-1004 retained a detectable PSII-associated band, whereas the PSII-associated band in PCC7806 was strongly diminished. To examine phycobilisome energy transfer, we recorded 77-K fluorescence emission spectra with 620-nm excitation, which predominantly excites phycocyanin in the phycobilisome. Under control conditions, C-1004 showed a similar distribution of phycobilisome-derived excitation energy to PSII and PSI, whereas PCC7806 showed relatively greater energy transfer to PSI (Fig. 5C). After 48 hours at 40°C, C-1004 maintained and even increased phycobilisome energy transfer to PSII at the expense of PSI, whereas PCC7806 continued to direct a greater share of phycobilisome excitation energy to PSI (Fig. 5D).

**Fig. 6.**
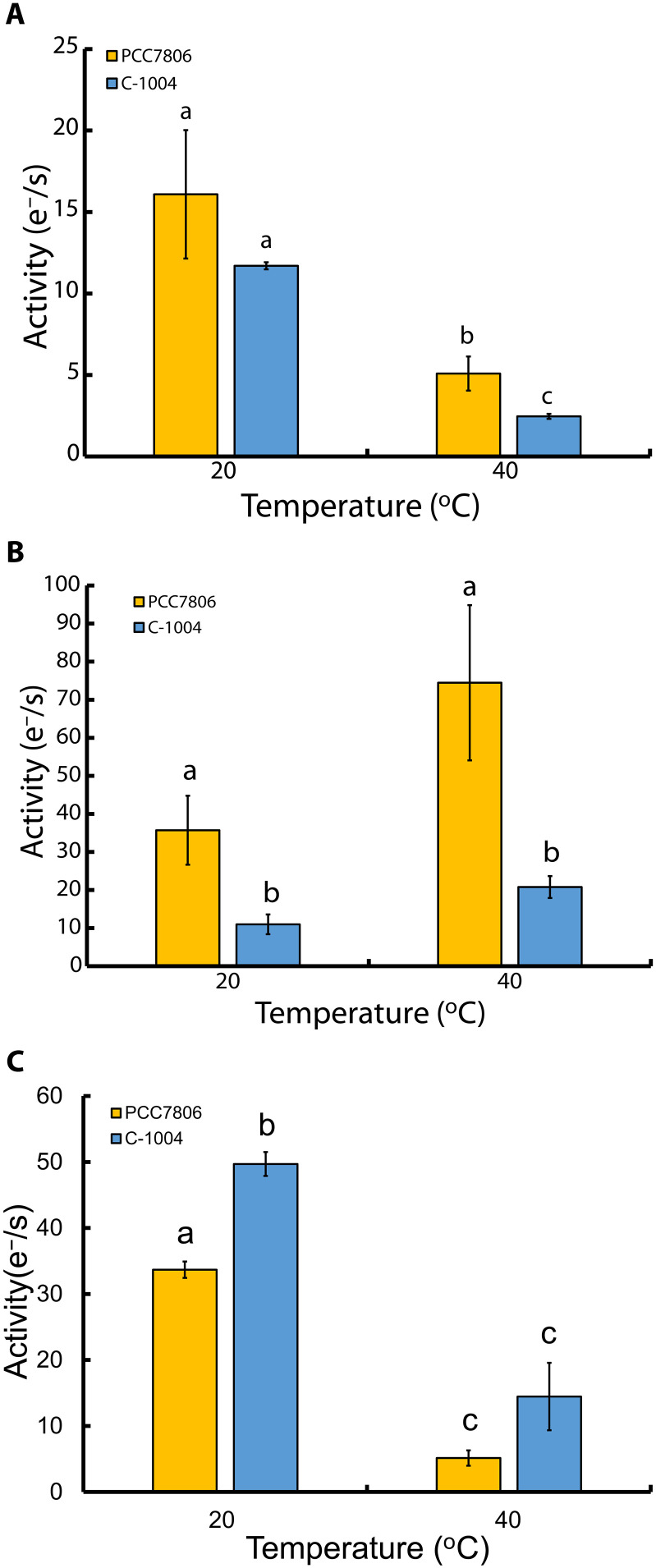
Heat stress differentially affects electron flow through b_6_f, PC, and PSI in PCC7806 and C-1004. Electron transport rates [electron per second (e^−^/s)] downstream of PSII in *M. aeruginosa* strains PCC7806 (yellow) and C-1004 (blue) at 20°C and after 48 hours at 40°C. Bars show means ± SEM (*n* = 3 biological replicates). (**A**) Cytochrome b_6_f complex. Electron transport rates through the b_6_f complex at 20° and 40°C. (**B**) PC. Electron transport rates supported by PC at 20° and 40°C. (**C**) PSI (P700^+^). Electron transport rates through PSI at 20° and 40°C. Within-strain changes with temperature were tested using repeated-measures ANOVA followed by paired two-tailed *t* tests. Between-strain differences at each temperature were tested using one-way ANOVA followed by independent-samples two-tailed *t* tests. Letters above the bars indicate groups that differ significantly across all comparisons (*P* < 0.05).

If PSII activity is retained in C-1004, even at reduced rates, then this should be reflected in downstream electron transport. We therefore measured electron flux through the cytochrome b_6_f complex, PC, and PSI (Fig. 6). At 48 hours, b_6_f-dependent rates were low in both strains, but PCC7806 remained ~2 times higher than C-1004 (*P* = 0.028, one-sided) (Fig. 6A). PC turnover was substantially higher in PCC7806, ~4 times at 20°C (*P* = 0.029, one-sided) and 3.5 times at 40°C (*P* = 0.012, one-sided) relative to C-1004 (Fig. 6B). However, PSI activity got reduced after 48 hours at 40°C [*P* = 0.007 (PCC7806) and *P* = 0.0174 (C-1004)]. The unchanged PC capacity in PCC7806 indicates that PC remains functionally active under heat stress, although this was not accompanied by a corresponding increase in PSI electron throughput at 40°C. This decoupling between PC and PSI electron transport in PCC7806 was further supported by a larger functional PC pool (Table 1). To quantify the functional concentration of these electron transfer components, we performed absorption measurements in the presence of 3-(3,4-dichlorophenyl)-1,1-dimethylurea (DCMU), methyl viologen (MV), and 2,5-dibromo-3-methyl-6-isopropyl-p-benzoquinone (DBMIB). These inhibitors promote maximal oxidation of the electron transfer complexes, allowing estimation of their maximal functional pool sizes. We found that the PC concentration in PCC7806 increased during prolonged heat stress, from 78.54 ± 22.87 nM at 20°C to 1303.33 ± 420.02 nM at 40°C (*P* = 0.05, one-sided, Cohen’s *d* = 1.86). In PCC7806, PC-dependent electron transfer capacity at 40°C was not reduced relative to 20°C, whereas PSI electron throughput decreased (Fig. 6B), suggesting that PC is unlikely to be the main bottleneck under these conditions.

**Table 1.** Functional concentrations of PSI, cytochrome b_6_f, and PC after extended heat stress. Functional concentrations of the main electron transport complexes downstream of PSII in *M. aeruginosa* strains PCC7806 and C-1004 at 20°C and after 48 hours at 40°C. Complex concentrations were estimated from maximum differential absorbance changes in the presence of specific photosynthetic inhibitors [3-(3,4-dichlorophenyl)-1,1-dimethylurea (DCMU), 2,5-dibromo-3-methyl-6-isopropyl-p-benzoquinone (DBMIB), and methyl viologen (MV)] using the following extinction coefficients: PSI, 70 mM^−1^ cm^−1^; b_6_f, 18 mM^−1^ cm^−1^; and PC, 4.7 mM^−1^ cm^−1^. Values are means ± SEM of three biological replicates.

Parameter	*M. aeruginosa* (PCC7806)		*M. aeruginosa* (C-1004)	
Temperature	20°C	40°C	20°C	40°C
PSI (nM)	31.47 ± 4.63^*^	83.61 ± 12.6^*^	50.1 ± 2.56	73.4 ± 14.89
*b_6_f* (nM)	33.91 ± 7.68^*^	607.33 ± 81.28^*^	29.53 ± 10.28^*^	1161 ± 323.89^*^
PC (nM)	78.54 ± 22.87^*^	**1303.33 ± 420.02** ^*^ ^†^	102.41 ± 39.05	126.97 ± 16.38^†^

* Statistically significant difference between temperatures within each strain.

† Statistically significant difference between strains.

**Fig. 7.**
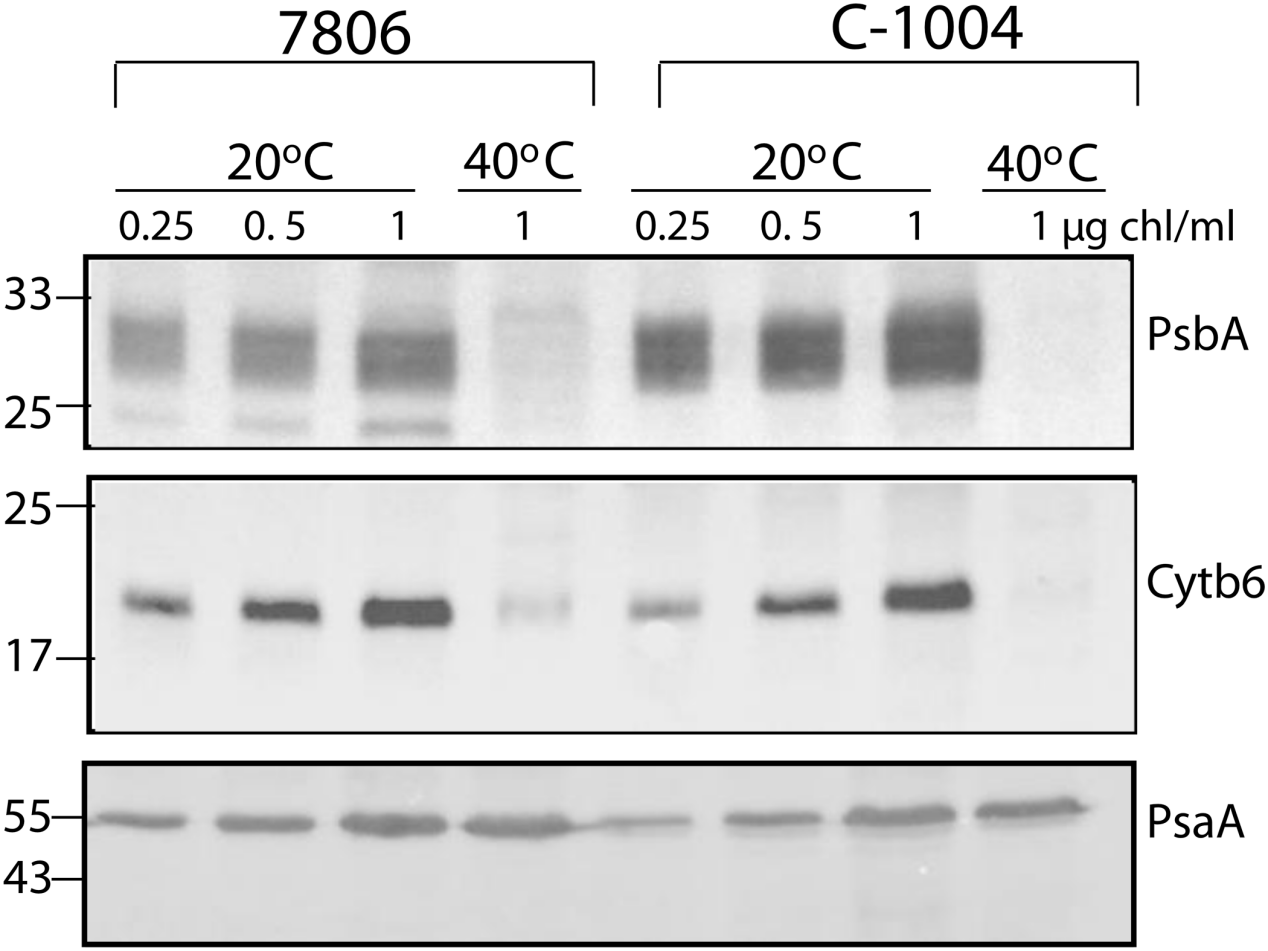
PCC7806 retains core photosynthetic proteins after extended heat stress, whereas C-1004 largely loses them. Western blot analysis of core proteins from the main photosynthetic complexes in *M. aeruginosa* strains PCC7806 and C-1004 at 20°C and after 48 hours at 40°C. The PSII reaction-center protein PsbA (D1), the cytochrome b_6_f core subunit cytochrome b_6_, and the PSI core subunit PsaA were immunodetected. Samples were loaded on an equal chlorophyll basis [microgram of chlorophyll per milliliter (μg chl/ml) per lane]. The blot shown is representative of two independent biological repeats.

To further assess the abundance of key photosynthetic complexes, we measured the protein levels of PsbA (PSII/D1), Cytb_6_ (cytochrome b_6_f), and PsaA (PSI) by immunoblotting (Fig. 7). At 20°C, C-1004 shows a slightly stronger band of PsbA than PCC7806 validating its faster activity at this temperature. At 40°C, the C-1004 band vanishes, whereas PCC7806 retains a faint band, consistent with overall PSII degradation at elevated temperatures. Bands of cytochrome b_6_ are strong in both strains at 20°C. They disappear at 40°C, completely in C-1004, with only a faint residual in PCC7806. This corroborates the overall lower b_6_f activity measured at 40°C and higher activity in PCC7806. Bands of PsaA are strong in both strains at both temperatures, consistent with a stable PSI core under this condition which is compatible with a contribution of electrons to a cyclic electron flow. An attempt to corroborate the increase in functional concentration of PC with PC antibody yielded unspecific cross reactivity which prevented its analysis.

**Fig. 8.**
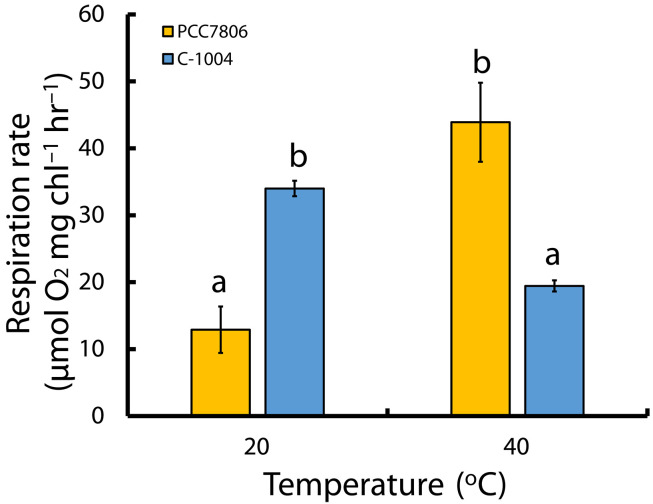
Extended heat stress reverses dark respiration rates: PCC7806 strongly up-regulates respiration while C-1004 is suppressed. Dark respiration rates (O_2_ uptake) of *M. aeruginosa* strains PCC7806 (yellow) and C-1004 (blue) measured by membrane inlet mass spectrometry (MIMS) at 20°C and after 48 hours at 40°C. The *y* axis shows dark O_2_ uptake rate; the *x* axis shows incubation temperature. Bars represent means ± SEM (*n* = 3 biological replicates). Within-strain effects of temperature (20° versus 40°C) were tested using repeated-measures ANOVA with Greenhouse-Geisser correction when sphericity was violated, followed by paired two-tailed *t* tests. Between-strain differences at each temperature were tested using one-way ANOVA followed by independent-samples two-tailed *t* tests (Welch’s *t* test when variances were unequal). Letters above the bars indicate groups that differ significantly across all comparisons (*P* < 0.05). chl, chlorophyll; hr, hour.

Although PC-dependent electron transport rates in PCC7806 did not decline after 48 hours at 40°C, PSI activity was, nevertheless, substantially reduced, despite abundant PSI in both strains. We therefore asked whether PC-carried electrons in PCC7806 were being redirected to an alternative sink. In cyanobacteria, downstream of the cytochrome b_6_f complex, COX competes with PSI for electrons transferred by PC and cytochrome c_6_ (*48*). To test whether the higher PC capacity was associated with greater respiratory electron flow, we measured dark O_2_ uptake using membrane inlet mass spectrometry (MIMS) (Fig. 8). Measurements in the dark isolate thylakoid respiration from light-driven photochemistry. Electrons enter the PQ pool via respiratory dehydrogenases (e.g., NDH-1 and SDH) and are consumed by terminal oxidases: CYD directly from PQH_2_ and COX downstream of b_6_f via c_6_/PC. Thus, dark oxygen consumption provides an integrated readout of respiratory flux through this pathway. At 40°C, respiration in PCC7806 increased to four times the rate observed at 20°C (*P* = 0.035) (Fig. 8). In contrast, respiration in C-1004 decreased by ~30% at 40°C (*P* = 0.047). This explains the increase in PC transport rate and functional quantity of PCC7806 after the extended heat stress.

**Fig. 9.**
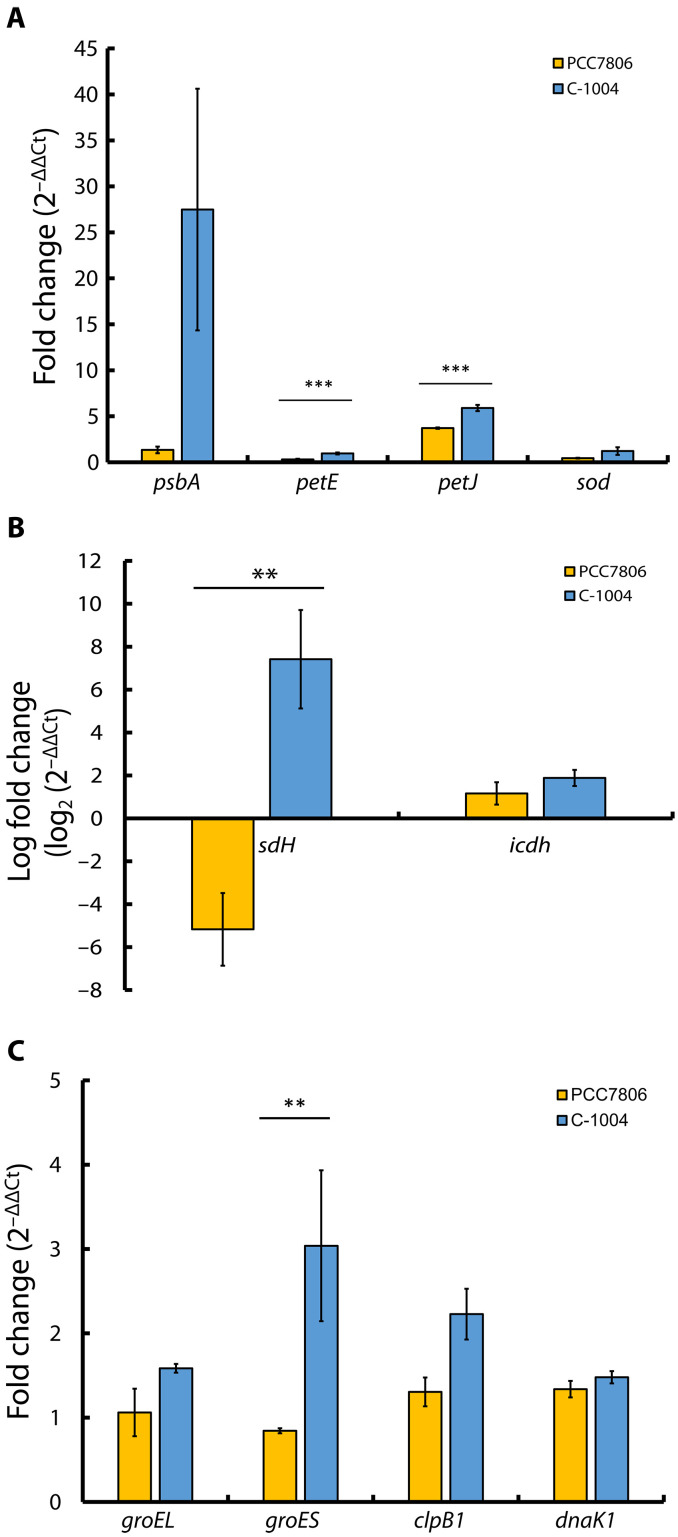
Extended heat stress induces distinct transcriptional responses in photosynthesis, respiratory, and heat-shock genes in *M. aeruginosa* PCC7806 and C-1004. Relative transcript levels (fold change) of selected genes in *M. aeruginosa* strains PCC7806 (yellow) and C-1004 (blue) after 48 hours at 40°C, normalized to their expression at 20°C. Bars represent the mean of three biological replicates (each based on three technical replicates) ± SEM (*n* = 3). Gene abbreviations: *groEL*, chaperonin 60; *groES*, co-chaperonin 10; *clpB1*, HSP100 disaggregase; *dnaK1*, Hsp70 chaperone; *psbA*, PSII D1 protein; *petE*, PC; *petJ*, cytochrome c_6_; *sod*, superoxide dismutase; *sdh*, succinate dehydrogenase; *icdh*, isocitrate dehydrogenase. Between-strain differences at each temperature were tested using one-way ANOVA followed by independent-samples two-tailed *t* tests (Welch’s *t* test when variances were unequal). (**A**) Photosynthesis-related and antioxidant genes. (**B**) Respiratory/TCA cycle genes. (**C**) Heat-shock and chaperone genes. Dots above the bars indicate groups that differ significantly across all comparisons (**P < 0.05; ***P < 0.01).

PCC7806 increased dark respiration and PC activity under extended heat stress, whereas C-1004 maintained PSII activity while lowering respiration, indicating a distinct coping strategy. To investigate the basis of these differences, we measured transcript abundance of PC (*petE*), cytochrome c_6_ (*petJ*), the D1 subunit of PSII (*psbA*), TCA cycle genes, and HSP targets (Fig. 9 and table S2). The soluble carrier cytochrome c_6_ (*petJ*) increased strongly in both strains during heat stress: from 2.10 ± 0.15 to 7.80 ± 0.47 in PCC7806 (*P* < 0.0001; table S2) and from 0.43 ± 0.03 to 2.49 ± 0.04 in C-1004 (*P* < 0.0001; table S2). Because cytochrome c_6_ is typically induced under stress, this pattern is consistent with both strains mounting a transcriptional stress response. Accordingly, in Fig. 9A, values greater than 1 indicate up-regulation at 40°C relative to 20°C, whereas values between 0 and 1 (as for *petE* in PCC7806) indicate reduced transcript abundance at 40°C. At the transcript level, *petE* (PC) did not show induction under heat stress: *petE* mRNA decreased in PCC7806 and remained approximately unchanged in C-1004 (table S2). Combined with the maintained, and possibly slightly enhanced, PC-mediated activity in PCC7806 (Fig. 6B), this pattern suggests that PC function under heat stress is sustained primarily through posttranscriptional regulation (as seen in enhanced of functional concentration in table S2) and protein stability rather than through transcriptional up-regulation of *petE*. PCC7806 showed a strong reduction in *sdh* transcripts under heat stress, from 0.22 ± 0.06 at 20°C to 0.02 ± 0.01 at 40°C (*P* < 0.01; table S2), whereas isocitrate dehydrogenase (*icdh*) transcripts decreased more modestly from 0.78 ± 0.22 to 0.67 ± 0.05 (*P* < 0.01; table S2). This pattern indicates reduced TCA-derived electron entry into the PQ pool in PCC7806 during prolonged heat stress. The regulation of *sdh* transcripts in PCC7806 is opposite to that observed in C-1004 (*P* = 0.016) (Fig. 9B). When inspecting the response of the HSPs gene transcripts, C-1004 increases its *groEL* transcripts from 0.55 ± 0.01 to 0.87 ± 0.01 (*P* < 0.001; table S2), and it increases its *groES* transcript substantially from 6.11 ± 0.91 to 17.00 ± 3.49 (*P* < 0.01; table S2). PCC7806 decreases its *groES* transcripts from 20.04 ± 0.41 to 16.91 ± 0.66 (*P* < 0.01). The difference in the fold change transcript level between the two strains is about 2 (*P* = 0.035, one-sided) (Fig. 9C). This implies for continuous stress in C-1004, which ultimately results in its reduced biomass. C-1004 maintained photosynthetic activity during extended heat stress but lost biomass, whereas PCC7806 survived while maintaining low photosynthetic activity. This contrast was further emphasized by the sustained high transcript levels of HSPs in C-1004 throughout the heat stress period. To place C-1004 in genomic context relative to PCC7806, we sequenced the C-1004 genome and performed a genome-wide comparison between the two strains. First, we compared their 16*S* ribosomal RNA (rRNA) sequences. A 16*S* rRNA distance tree (fig. S1) places both strains within the *M. aeruginosa* cluster, with ~99% sequence identity between PCC7806 and C-1004. Consistent with this, the two genomes have very similar sizes and nucleotide compositions [PCC7806: 5,139,339 base pairs (bp), 42.09% Guanine-Cytosine content. (GC); C-1004: 5,069,526 bp, 42.30% GC; table S3]. *K*-mer–based genome metrics further indicate highly similar overall composition and sequence content. Data suggest size similarity of 98.64%, composition similarity of 99.90%, *k*-mer Jaccard similarity of 100%, and *k*-mer cosine similarity of 99.92% (table S4). At the same time, the direct sequence similarity across the full alignment is more modest (25.75%, overall similarity score of 77.52%; table S4). A whole-genome alignment with Mauve (*49*) (fig. S2) reveals a largely conserved chromosomal backbone interspersed with several strain-specific genomic blocks and local inversions. Genome-based digital DNA-DNA hybridization (dDDH) values obtained from Type Strain Genome Server (TYGS) (*50*) [d0 = 87.1%, d4 = 81.7%, and d6 = 89.1%, G+C difference of 0.21% (table S5)] confirm that PCC7806 and C-1004 are conspecific *M. aeruginosa* strains. Genome BLAST Distance Phylogeny (GBDP) phylogeny tree (*51*) places them as a closely related sister pair within the *M. aeruginosa* clade, distinct from other *Microcystis* genomes included in the analysis (fig. S3). Scanning for Rre1-like consensus motifs (table S6) showed promoter-proximal sites in C-1004 at several canonical heat-shock genes. These included *clpB*, *groEL*, *groES*, *dnaJ*, *hsp70*, and *hsp20*, all within roughly 400 bp upstream. Rre1-like motifs in C-1004 also appeared near TCA cycle genes, including citrate synthase, *icdh*, and *sdh*. Additional sites were detected upstream of multiple photosynthetic and electron-transfer loci, such as *psaC*, *psaD*, *psaK*, *psbX*, and *psbY*. Motifs also occurred at *petA*, encoding cytochrome f, and several Fd-related genes involved in light-driven electron transport. In PCC7806, Rre1-like motifs were present upstream of heat-shock chaperones, including *clpB* and *dnaK* or *dnaK2*. Among the surveyed PCC7806 genes, motifs were not detected at TCA cycle loci but concentrated at photosystem components. These included PSII D2 and CP43, several PSI subunits such as *psaK2* and *ycf3*, and other electron-transfer proteins. This distribution broadly parallels the transcript data, where C-1004 shows stronger late HSP and TCA induction at 40°C. Conversely, PCC7806 displays more limited late up-regulation of these pathways under the same prolonged heat-stress conditions.

## DISCUSSION

**Fig. 10.**
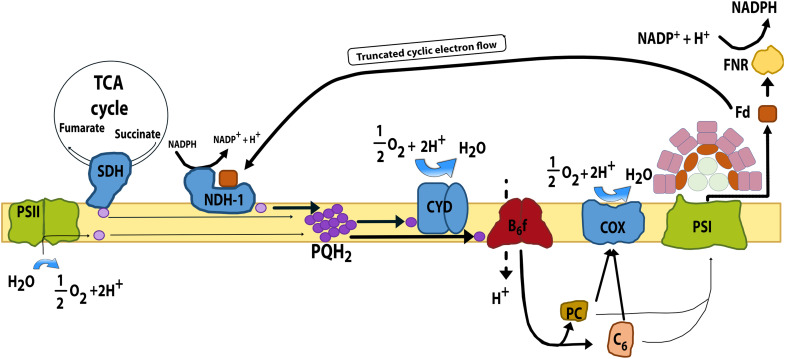
Respiratory electron bypass compensates for photosynthetic impairment under extended heat stress. Schematic representation of the proposed electron transport configuration in *M. aeruginosa* PCC7806 after 48 hours at 40°C, when PSII activity is strongly reduced or lost. In the model, we depict the phycobilisome as functionally reallocated from PSII to PSI, not a physical migration, but a shift in excitation energy transfer that preferentially feeds PSI. Electrons are then injected into the thylakoid chain primarily via Fd and FNR. Reduced Fd and NADPH donate electrons to the NDH-1 complex, which, in turn, feeds the PQ (PQ/PQH_2_) pool. From there, electrons pass through the cytochrome b_6_f complex and on to terminal oxidases (CYD and COX) rather than returning to PSI, hence, a truncated cyclic electron flow. This configuration represents a truncated form of cyclic electron transport around PSI, in which PSI and NDH-1 sustain electron flow, but a substantial fraction is diverted to respiratory sinks instead of completing a full PSI-to-PSI cycle.

In controlled laboratory conditions, we compared two closely related strains of *M. aeruginosa* under extended heat stress that approaches physiological limits. The Kinneret isolate C-1004 tended to maintain photosynthetic activity while respiration lagged, and the culture ultimately lost biomass. In contrast, the reference strain PCC7806 down-regulated PSII activity, increased respiratory electron flow, and persisted for longer. Integrating the molecular, biochemical, and functional readouts, we propose a working model of a truncated cyclic electron transport route (Fig. 10): During extended heat stress, *M. aeruginosa* can partly compensate reduced PSII activity. Electrons are excited by PSI and are transferred directly to Fd or transferred by Fd to FNR to produce NADPH. Fd and NADPH are transferring electrons to NDH-1 (or equivalents). These are transferred by the PQs and are consumed by quinol oxidases or pass through b_6_f and PC and are consumed by terminal oxidases. This rerouting may keep PSI sufficiently oxidized to remain functional while sustaining a trans-thylakoid proton-motive force and a modest NADPH supply, thereby supporting survival in PCC7806.

Cyanobacteria are known to modulate respiration in response to various environmental stresses, for example, as a response to high salinity (*52*), cold temperatures (*53*), and high light intensities (*54*). The most common modulation is a shift from linear to cyclic electron flow via the NDH-1 complex, which serves both photosynthetic and respiratory roles in cyanobacteria (*15*). In our experimental conditions, the data are consistent with PCC7806 not restoring PSII D1 transcript and instead engaging the truncated cyclic, respiration-coupled route. In C-1004, despite increased PSII transcripts, an overall PSII:PSI balance, and induction of HSP genes, these adjustments were insufficient to prevent a reduction in biomass.

The 77-K fluorescence profiles support the distinct energy-allocation strategies suggested for the two strains under extended heat stress. Under control conditions at 20°C, C-1004 showed a higher PSII-associated fluorescence band at 680 nm than PCC7806 (Fig. 5A, blue peak), consistent with its higher maximal electron transport rates (Fig. 4A, blue curve). After 48 hours at 40°C, PCC7806 showed an almost complete loss of the PSII-associated band at 680 nm (Fig. 5B, yellow peak), corroborating the strong suppression of PSII activity observed in the RLCs (Fig. 4B, yellow curve). In contrast, C-1004 retained a clear PSII-associated band at 40°C (Fig. 5B, blue peak at 680 nm), consistent with its sustained PSII activity (Fig. 4B, blue curve) and with the increased allocation of excitation energy to PSII under heat stress (Fig. 5D, blue peak at 680 nm).

At the same time, the PSI-associated fluorescence band at 710 to 730 nm remained pronounced in both strains under both temperature conditions (Fig. 5, A and B) and, in all cases, remained higher than the PSII-associated band. This pattern is consistent with the stable PsaA protein abundance observed by immunoblotting (Fig. 7). However, PSI activity declined under heat stress (Fig. 6C), indicating that maintenance of PSI abundance did not translate into maintained PSI electron throughput. In C-1004, continued energy transfer to PSII together with reduced PSI activity may create a bottleneck at the cytochrome b_6_f complex, increasing photosynthetic stress and contributing to strain degradation. By contrast, PCC7806 maintained excitation energy directed mainly toward PSI at both 20° and 40°C (Fig. 5, C and D, respectively, yellow curve) while retaining only minimal PSII activity (Fig. 4B). Together with the sustained PC capacity (Fig. 6B) and increased dark respiration (Fig. 8), this suggests that PCC7806 reroutes electrons toward respiration despite reduced PSI activity. This rerouting may compensate for impaired photosynthetic electron transport and support survival under prolonged heat stress.

When interpreting the functional concentration of the b_6_f complex (Table 1), it can be seen that the functional concentration increases for both strains (Table 1). However, it decreases in activity (Fig. 6) and Western analysis (Fig. 7). The use of mix of inhibitors drives the electron transport chain to defined redox states and can amplify cytochrome b_6_f signals even when Q_o_-site turnover is blocked (*55*); thus, an increased optical amplitude can co-occur with lower enzymatic throughput. Loss or damage of the Rieske Fe-S center (or other assembly defects) would leave redox enabled heme present but disable Q-cycle catalysis. This aligns the spectroscopy increase in functional concentration with reduced electron transport rate and complex density in Western under extended heat stress. Practically, the level of b_6_f complex and flux provide the correct readout of capacity in our experimental conditions, while inhibitor-defined amplitudes reflect residual redox pools, as suggested before (*56*).

Promoter mapping in both *Microcystis* genomes points to candidate Rre1 control points, but their placement spans different functional targets in each strain. Our motif survey (table S6) is consistent with the Hik34-Rre1 module activating heat-inducible chaperone and stress promoters in cyanobacteria (*33*). In C-1004, the higher density of putative Rre1 sites near HSP genes (e.g., *clpB*, *groESL*, *hsp70*, and *hsp20*) and TCA nodes (e.g., *gltA*, *icdh*, and *sdh*/*frd*) is consistent with its stronger 48 hours up-regulation of HSP and TCA transcripts under extended heat stress (Fig. 9). This pattern is compatible with reports that prolonged warming can sustain or reconfigure stress and central-metabolism programs in *Microcystis* (*57*). By contrast, PCC7806 has relatively few putative Rre1 sites near heat-shock genes and correspondingly shows little late (48 hours) HSP up-regulation (*58*). Together, these observations suggest that Hik34-Rre1 signaling is present in both genomes but distributed differently across HSP, TCA, and electron transport chain genes. This assists in explaining C-1004’s sustained late HSP/TCA response versus more transient HSP dynamics in PCC7806. Last, with regard to the possibility that additional respiratory complexes found on the cytoplasmic membrane were involved in the increased respiration during the shock (*16*), we found no evidence of a conserved ARTO in the genomes of *Microcystis* PCC7806 and C-1004. BLAST analysis (*59*) returned only low-identity matches (34.9% identity and 79% coverage), suggesting that, if ARTO-like sequences are present, then they are unlikely to be functional. Therefore, respiration in *Microcystis* is most likely restricted to the thylakoid membrane and the combined photosynthesis/respiration electron transport chain.

Our experimental temperature of 40°C is supraecological for Lake Kinneret, but it can be viewed as an upper boundary along a warming continuum that is already emerging in some freshwater systems. During the 2023 Amazon drought and heat wave, several shallow lakes exceeded 37°C during the day, and one ~2-m-deep lake reached up to 41°C throughout the entire water column, on top of a long-term regional warming trend (*44*). Such events illustrate that temperatures approaching or even exceeding the thermal tolerances of aquatic organisms can already occur during extreme conditions. Laboratory studies of freshwater *Microcystis* strains are consistent with this picture. Tropical bloom-forming strains show higher biomass at 31° to 35°C compared to 27°C, where most strains still grow at 37°C (*60*). Growth and buoyancy of *M. aeruginosa* also respond strongly to temperature across a broad range from 5° to 35° C (*61*). Together, these field and laboratory observations indicate that some *Microcystis* populations already operate close to their upper thermal range under present and near-future climate conditions. In this context, our 40°C treatment is best interpreted as an upper boundary scenario along this warming continuum rather than as a typical present-day condition in Lake Kinneret. By pushing cells close to their thermal failure point, the experiment reveals how the balance between photosynthesis and respiration shifts near the upper thermal limit. This mechanism is likely to become important under less extreme but recurrent or prolonged warming events. Framed this way, our results suggest that rerouting electrons toward respiration during extended heat stress may contribute to survival near the upper thermal limit. Consistent with this, PCC7806 maintained respiration and survived the challenge, whereas C-1004 did not.

This study has several limitations. First, because *M. aeruginosa* C-1004 is a nonaxenic field isolate, contributions from a low-abundance associated bacterial community cannot be completely excluded. However, cultures were cultivated in BG-11 mineral medium (*62*, *63*) without added organic carbon, which favors cyanobacterial photoautotrophic growth and is relatively poor for heterotrophic bacteria (*64*, *65*). Second, both strains were maintained under long-term laboratory culturing, including continuous light conditions, before the experiments. Given the documented diel transcriptional regulation and physiological plasticity of *M. aeruginosa*, such maintenance may influence strain-specific properties (*66*). Therefore, while the mechanistic differences observed here remain informative, broader ecological interpretation of the strain-specific responses should be made with caution. Third, while our focus was on the thylakoid-associated respiratory complexes, cyanobacteria also have other oxygen-consuming proteins that may contribute to oxygen dissipation under stress. In particular, flavodiiron proteins (Flv1 and Flv3) catalyze photoreduction of oxygen without generating reactive oxygen species and are key to balancing the redox state during photosynthesis (*67*). Although we did not assess Flv involvement during the extended heat stress, their known role suggests they could complement or substitute for the pathways that we studied. Fourth, the absence of ^18^O_2_-based MIMS measurements, could directly resolve respiratory activity in the light. However, interpretation of such measurements in cyanobacteria is complicated precisely by Flv activity, which can obscure canonical respiration. Resolving this would require Flv mutants, which were not available for the *Microcystis* strains used in this study. We therefore relied on dark respiration and complementary physiological measurements. These nevertheless consistently supported our conclusion that respiration compensates for the decline in photosynthesis during extended heat stress. Fifth, we did not perform direct viability assays (e.g., live/dead flow cytometry); thus, cell density serves as a population-level survival proxy. Future work will incorporate viability staining or plating to distinguish live and dead subpopulations under heat stress. Sixth, we did not directly measure nonphotochemical quenching (NPQ) in *Microcystis*. Although we confirmed the presence of an orange carotenoid protein (OCP) (*68*) homolog in both PCC7806 and C-1004 (81% identity to *slr1963* of *Synechocystis* PCC6803), quantifying OCP-dependent NPQ requires specialized setups. Specifically, (i) OCP activation requires strong blue-green light and produces spectral signatures that are difficult to capture with our instruments (*68*), and (ii) separating OCP-mediated quenching from other dissipative processes typically requires mutants that are now unavailable for *Microcystis* C-1004 and high-resolution time-resolved spectroscopy (*69*). Therefore, we focused instead on parameters directly accessible in our setup while acknowledging the role of OCP-dependent NPQ as an important photoprotective mechanism in cyanobacteria. Seventh, we did not quantify respiratory substrates such as glycogen or soluble sugars, which would require dedicated chromatographic analysis. Although our gene expression data suggest an up-regulation of the TCA cycle in C-1004, it eventually did not result in increased respiration. On the contrary, *b_6_f* complex activity was reduced, implying for an excess reduced electron transport “jam” upstream to the *b_6_f* complex*.* This may have increased the photosynthetic stress, as C-1004 eventually lost biomass. Future work should incorporate glycogen and sugar quantification to better constrain the substrate supply driving respiratory flux. Last, heat-shock transcripts in cyanobacteria typically spike rapidly (∼15 to 60 min) after a temperature upshift (*26*, *31*); this early, transient induction has been documented for *groESL*, *htpG*, *hspA*, *clpB1*, and others. Because our measurements were performed at the late phase of 48 hours, these measurements capture late outcomes and cannot resolve this induction window. Future work should include ≤1- to 3-hour time courses and tests of Hik34/Rre1-dependent control to quantify the immediate transcriptional response in *Microcystis*.

Under a temperature step upshift to 40°C with 48-hour exposure, PCC7806 and C-1004 show contrasting physiological responses. Although PSI and *b_6_f* complexes electron transport rates are minimal in both strains at 48 hour, PCC7806 maintains viability, whereas C-1004 declines in biomass. Together, the measurements suggest that PCC7806 maintains energetic balance via increased dark respiration, whereas C-1004 prioritizes photosynthetic repair with limited respiratory compensation. These patterns suggest that respiration can act as a compensatory route when photosynthetic throughput is decreased. This study marks the value of strain-level comparisons for anticipating cyanobacterial responses to warming. While suggesting NDH-1 as the complex through which electrons are reentering the electron transport chain, we do not pinpoint the electron sources that support increased respiration in PCC7806; resolving these, along with early heat-response kinetics and protein-level regulation, will require targeted follow-up to refine the mechanistic picture.

## MATERIALS AND METHODS

### Cell cultures

*M. aeruginosa* PCC7806 was provided to the Kinneret Limnological Institute by A. Kaplan, Hebrew University. *M. aeruginosa* C-1004 is an isolated Lake Kinneret strain by O. Hadas (*70*) and is maintained and available on request at the Israeli National Culture Collection of Algae (*71*, *72*). Both strains were cultivated in BG-11 medium in incubators at 20°, 24°, or 32°C in 125-ml flasks (*63*). Cultures were grown on an orbital shaker at 100 rpm. Cultures were illuminated continuously by warm white light from light-emitting diodes at an intensity of 35 μmol photons m^−2^ s^−1^. No diurnal light/dark cycle was applied during acclimation or experimental growth. For acclimation, cultures were maintained by serial batch transfers at the respective temperatures (20°, 24°, or 32°C). Each cycle consisted of growth under exponential conditions until reaching the stationary phase, followed by dilution into fresh BG-11 medium (*63*). This procedure was repeated for at least 10 sequential growth cycles, corresponding to about 10 months at 20°C. At higher temperatures, cycles covered a shorter total period but were still sufficient to ensure stable acclimation before measurements. Cultures were tracked for growth using a standard hemocytometer.

### Extended heat stress

For heat-shock experiments at 40°C, two water baths were prepared with 20° and 40°C constant temperatures. Samples from mid-log phase cultures at 20°C were replaced with fresh BG-11 medium and were placed in vertical glass tubes, sparged with air in the baths at the same starting chlorophyll concentration, which was 10 μg/ml. Illumination came from the side at the same light qualities and intensities as experienced during cultivation. The experiment lasted 48 hours after which samples were taken for chlorophyll a analysis and ecophysiological measurements or were frozen at −70°C until use in the differential expression procedure or spectroscopic analysis.

### Chlorophyll a analysis

Each replicate (1 ml) was centrifuged at room temperature at 6800*g* for 1 min and was replaced with 90% acetone of American Chemical Society grade (Chen-Shmuel Chemicals, Haifa, Israel). Then, each replicate was gently mixed by pipetting in the dark and on ice. Samples were then incubated at 4°C overnight and then were vortexed and centrifuged at 12,300*g* to remove impurities from the extracted pigments. Readings were acquired with a UV-VIS spectrophotometer (Uvikon XS, Secomam, France) at 664 nm (*73*).

### Ecophysiological measurement preparations

When preparing for ecophysiology measurements in a metabolic chamber, samples were replaced with fresh BG-11 medium with 50 mM Hepes (Sigma Aldrich, St. Luis, Missouri, USA) and 1 mM final concentration of dissolved inorganic carbon (NaHCO_3_, Sigma Aldrich, St. Luis, Missouri, USA). Samples for analysis from either 20° or 40°C were placed in a metabolic cuvette holder connected to a Joliot Type Spectrophotometer (JTS-150, Spectrologix, Knoxville, Tennessee, USA). Temperature was set by a water bath (Thermo Fisher Scientific, Waltham, Massachusetts, USA) with circulating water in the walls of the metabolic chamber at 20°C for all experiments. Water was circulated for at least half an hour before the tests to stabilize the temperature within the chamber. The metabolic chamber held a two-sided quartz cuvette, either opened or sealed with a septum plug (CotsLab, London, UK), depending on the measurement technique. Samples were mixed with a Teflon microstirrer at 100 rpm within the quartz cuvette. Samples were dark adapted for at least 5 min to allow the photosynthetic apparatus to relax and to reach its typical maximum quantum yield fluorescence in the dark [for cyanobacteria, Fv/Fm (the ratio of variable fluorescence to maximum fluorescence) = ~0.45; (*74*)].

### Light response curves

RLCs (*75*) were measured with a custom protocol on the JTS instrument, with 10-s illumination at actinic light intensities of 10 to 2400 μmol photons m^−2^ s^−1^ (620 nm). From these measurements, we derived rETR, calculated as the product of effective PSII quantum yield and incident light intensity. We note that we did not determine absolute ETR (*76*), which requires absorption cross-sectional estimation from fluorescence rise kinetics (*77*). At the end of each period, an effective quantum yield measurement was acquired with a saturating pulse of 700 ms (*78*). For each strain and temperature, RLCs were then fitted with the three-parameter empirical Eilers and Peeters photosynthesis-irradiance model (*79*). From these fits, we extracted the curve parameters: initial slope [light use efficiency in arbitrary units (a.u.)], optimum intensity [*I*_m_; micromoles of photons per square meter per second (μmol photons m^−2^ s^−1^)], characteristic intensity (*I*_k_; μmol photons m^−2^ s^−1^), and maximum photosynthesis (Pm; a.u.).

### 77 K excitation fluorescence analysis

Samples from 20° and 40°C were stored at −70°C until analysis. Before measurement, samples were thawed on ice and aliquoted into Pasteur pipettes, which were then immersed in liquid nitrogen in a Dewar flask inside the spectrofluorometer. Fluorescence spectroscopy (77 K) was performed using a QuantaMaster spectrofluorometer (PTI, Horiba, Kyoto, Japan). Emission spectra were recorded from 650 to 760 nm with both excitation and emission slit widths set to 5 nm. For preprocessing and analysis, spectra were restricted to the 670- to 740-nm interval to exclude edge-region artifacts. Under both 435- and 620-nm excitation, the 650- to 670-nm region showed a sloping baseline that interfered with interpretation of the emission profiles, whereas the 740- to 760-nm region contained apparent far-red peaks that were considered instrumental artifacts. Restricting the analysis window improved the clarity and comparability of the spectra. Each spectrum represents the average of three consecutive scans to improve the signal-to-noise ratio. For spectra recorded with 620-nm excitation, baseline correction was applied using an exponential regression trend to better resolve the relevant peaks. Spectra were plotted on a common intensity scale rather than normalized to maximal emission to preserve biologically relevant differences in emission magnitude between strains and treatments.

### Intersystem electron transport rates and functional concentrations

Measurements of the electron transport rate through the b_6_f, PC, and PSI complexes were carried out using the dark pulse technique (*80*) with the respective filters: BG-39 for the *b_6_f* and PC acquisition and 705-nm filter for the PSI acquisitions (P700). For each of these measurements, fresh sample from the replicates experiencing heat shock or controls were taken for analysis, except in the case of analyzing the functional concentrations of the units. In this case for each acquisition, readings without and with addition of herbicides DCMU (D2425, Sigma-Aldrich, St. Luis, Missouri, USA), MV (36541, Sigma-Aldrich, St. Luis, Missouri, USA), and DBMIB (271993, Sigma-Aldrich, St. Luis, Missouri, USA) were acquired. Rereduction rates of *b_6_f*, PC, and PSI were calculated from the half-time of the rise of the rereduction of each complex (*56*).

### Western analysis

Cells were harvested by centrifugation at 3500*g* for 10 min. The cells were then resuspended in TMN buffer [50 mM tris (pH 7.5), 10 mM NaCl_2_, and 5 mM MgCl_2_ supplied with antiprotease cocktail] (cOmplete Mini, EDTA-free; Merck, 11836170001). Cell lysis was performed using a cell disruptor (CF1, Constant Systems, Daventry, UK) at 30,000 psi. The lysate was centrifuged at 3000*g* for 5 min, and the supernatant was collected and further centrifuged at 17,000*g* for 30 min. Chlorophyll concentration of the resulting pellet was determined by measuring in samples extracted in 100% methanol. Absorbance was measured using a spectrophotometer (UV-1900i, Shimadzu, Kyoto, Japan) at 665 nm (*81*). Thylakoids at final chlorophyll concentration of 1 μg/ml were resuspended in Laemmeli’s SDS-sample buffer (*82*). Following separation on 14% acrylamide gel, proteins were transferred to polyvinylidene difluoride membrane and exposed to three different antibodies against: PsbA (Agrisera, catalog no. AS05084), PsaA (Agrisera, catalog no. AS06172), and cytochrome b_6_ (Agrisera, catalog no. AS184169). WesternBright ECL kit (Advansta, catalog no. K-12045-D50) was used as chemiluminescence substrate. The signal was captured using Gel Imager (ChemiDoc, Bio-Rad, Hercules, California, USA).

### Gas exchange

Gas exchange was measured in septum-sealed, two-sided quartz cuvettes by a custom-constructed MIMS (Pfeiffer Vacuum, Aßlar, Germany) with a silicone tube used as a nose, as described before (*83*, *84*). Light intensity for the rereduction and gas exchange analyses was set at 1200 μmol photons m^−2^ s^−1^, as at this intensity the cultures at 20°C reached their maximum PSII activity (see Fig. 4 for visual explanation).

### Differential gene expression analysis

RNA extraction used a combined approach, integrating optimal TRIzol methods from two sources for enhanced efficacy (*85*). TURBO deoxyribonuclease (2 U/μl) from Invitrogen (catalog no. AM2238) was used in the process as well. Harvested cyanobacteria samples were transferred to 2-ml O-ring Eppendorf tubes, centrifuged, and amended with 500 μl of TRIzol (15596026, Invitrogen, Waltham, Massachusetts, USA) and were pipetted for thorough gentle mixing. Then, 50 μl of Proteinase K (SAE0151, Sigma-Aldrich, St. Luis, Missouri, USA) was added, and the mixture was gently mixed and incubated for 2 hours at 56°C. Bromochloropropane (100 μl; B9673, Sigma-Aldrich, St. Luis, Missouri, USA) was added and gently shook until homogenous, and samples were incubated at room temperature for additional 10 min. This step was repeated twice, and then 500-μl RNA fraction was added with 400 μl of ethanol 70% (Chen-Shmuel chemicals, Haifa, Israel) and 100 μl of sodium acetate (S8750, Sigma-Aldrich, St. Luis, Missouri, USA). Replicates were let incubate for 10 min at room temperature. We then followed a standard RNA extraction by commercial kit (RNeasy, QIAGEN, Hilden, Germany). RNA concentration was assessed through absorbance measurements at 260 nm using the NanoDrop 2000c Spectrophotometer (Thermo Fisher Scientific, Waltham, Massachusetts, USA). The evaluation of RNA purity and integrity involved examining A260/280 and A260/230 ratios derived from spectrophotometric readings. RNA (1 μg) from all treatments was reverse transcribed into cDNA using the UltraScript cDNA synthesis kit (PCR Biosystems, Wayne, Pennsylvania, USA) following the manufacturer’s instructions. Reaction conditions were as follows: 42°C for 30 min, followed by 85°C for 10 min. Gene expression was measured using real-time quantitative polymerase chain reaction (qPCR; StepOnePlus Real-Time PCR System, Thermo Fisher Scientific, Waltham, Massachusetts, USA). The experiments were carried in triplicates for each reaction, and negative controls were performed on the RNA extractions to ensure no genomic DNA contaminations. Reactions were carried in Ultra Clear qPCR Caps (catalog no. AB0866) and the use of qPCRBIO SyGreen blue mix Hi-ROX (PCR Biosystems, Wayne, Pennsylvania, USA). Reaction conditions were as follows: 95°C for 2 min (1 cycle), followed by 95°C for 5 s, 60°C for 20 s, 95°C for 15 s, 60°C for 1 min, and 95°C for 15 s (40 cycles). Changes in gene expression were measured at different treatments and time points. Relative expression of genes was calculated by the comparative threshold cycle method method (*86*). Phosphoenol pyruvate carboxylase gene was used as a housekeeping gene for normalization of RNA quantity (*87*). Primers were designed using BLAST (*59*) and BioEdit software, followed by validation using OligoAnalyzer on the IDTdna website (https://eu.idtdna.com/page). Subsequently, the primers underwent testing through PCR and gel electrophoresis to ensure both size validation and specificity. List of primers are shown in table S7.

### Genome sequencing

Genomic DNA from a C-1004 culture was extracted for genomic sequencing using a Zymbiomics DNA miniprep kit (Zymo Research, Irvine, California, USA). Illumina shotgun short-read libraries were prepared following the KAPA EvoPlus V2 kit protocol (Roche, Basel, Switzerland) together with a KAPA library amplification kit. Briefly, 500 ng of input DNA was fragmented, A-tailed, adapter ligated, and bead cleaned. Then, four cycles of KAPA library amplification were performed on 25 μl of cleaned library. Sequencing was performed on an Illumina NovaSeq X instrument with 2 × 150 base reads. A long-read shotgun library was prepared with a LongPlex Long Fragment Multiplexing Kit (seqWell, Massachusetts, USA) using a Whole Genome Sequencing (WGS) PCR-free protocol. The resulting library was sequenced on PacBio Revio (Menlo park, California, USA), generating reads mostly in the range of 3 to 15 kb. Library preparation was performed at the Genomics and Microbiome Core Facility at Rush University, and sequencing was performed at the Roy J. Carver Biotechnology Center at the University of Illinois at Urbana-Champaign. Raw sequence data files were submitted in the Sequence Read Archive of the National Center for Biotechnology Information (NCBI). The BioProject identifier of the samples is PRJNA1268147, and the Biosample identifier of the sample is SAMN48743338

### Genome assembly and annotation

De novo assembly of the C-1004 genome was performed within the software package CLC Genomics Workbench (CLC v25; QIAGEN, Hilden, Germany) using the long-read assembler with PacBio long-read data. Bacterial whole-genome de novo assembly was performed on PacBio sequence data after trimming raw data for quality and length. The following conditions were used to prepare the data, including automatic removal of adapter sequences, removal of data quality below Q20, removal of sequences with >2 ambiguous nucleotides, removal of homopolymers, and removal of sequences shorter than 3000 bases. After trimming of the initial 793,957 sequences, 782,054 sequences remained for assembly and the mixed microbial community assembled into 874 contigs between 4491 and 5,069,426 bp (N50 = 158,634 bases). The average length of sequences after trimming was 5273 bases. The long contig (Utg75812) was further annotated and analyzed for genome completeness.

The software package Prokka (v1.14.6) was used for genome annotation of the C-1004 sequenced genome. Prokka is a rapid and efficient tool for the annotation of prokaryotic genomes, using a variety of well-established databases such as UniProt, RefSeq, and Pfam (*88*). It predicts genes and their functions and identifies features such as rRNAs, tRNAs, and signal peptides. The genus *Microcystis* was specified for Utg75812, and this designation allowed Prokka to reference appropriate databases for the prediction. CheckM (v1.1.3) (*89*) was used to verify genome completeness and contamination. A lineage-specific workflow was run to generate a comprehensive report on genome completeness, contamination, and strain heterogeneity.

### Genome-wide comparisons

To compare PCC7806 and C-1004 at the genomic level, we combined 16*S* rRNA phylogeny, basic genome descriptors, whole-genome alignment and genome-based distance metrics. 16*S* rRNA gene sequences from the following *M. aeruginosa* strains were retrieved from NCBI for phylogenetic comparison (*59*): PCC7806 (CP155078.1), NIES-843 (NR_074314.1), PCC7941 (U40340.2), NIES-88 (U40337.2), and C-1004 (sequenced genome). We analyzed the two cyanobacterial genomes with a small Python workflow. FASTA files were validated and parsed, and genome-scale descriptors (total length, GC%, and contig count) were computed with custom scripts built on Biopython (SeqIO) (*90*). Base-composition profiles (A, T, G, and C fractions) were calculated from the parsed sequences.

Genome-wide sequence similarity between *M. aeruginosa* strains C-1004 and PCC7806SL was assessed using *k*-mer frequency analysis with *k* values ranging from 3 to 6 nucleotides. For each genome, all possible *k*-mers were extracted using a sliding-window approach, excluding *k*-mers containing ambiguous bases (*N*). Jaccard similarity coefficients were calculated as the ratio of shared *k*-mers to the total number of unique *k*-mers between genomes (*91*), whereas cosine similarity was computed from *k*-mer frequency vectors (*92*).

Whole-genome alignment between PCC7806 and C-1004 was performed with Mauve (*49*) using the progressive alignment algorithm with default parameters. Locally collinear blocks were identified and visualized to assess conserved backbone regions, strain-specific genomic islands, and inversions.

For genome-based taxonomy and phylogeny, we submitted the PCC7806 and C-1004 genomes to the TYGS (*50*, *93*). dDDH values were calculated using the GBDP method (formulas d0, d4, and d6) (*51*). A GBDP tree was inferred from intergenomic distances with branch support values (*94*), rooted at midpoint (*95*), and visualized with PhyD3 (*96*).

### Regulatory motif search

To identify potential cis-regulatory elements of the Hik34-Rre1 heat-shock system, we scanned promoters of *M. aeruginosa* PCC7806 (GCF_002095975.1_ASM209597v1, NCBI) and C-1004 (SAMN48743338) for the short Rre1 consensus (GTnCGG[t/g]) (*33*). Upstream promoter regions (500 bp) were extracted for all protein-coding genes, with strand orientation taken into account. Target genes were classified into four functional categories: photosynthesis light reactions and electron transport, terminal and quinol oxidases in respiration, TCA cycle enzymes, and HSPs, based on gene product descriptions and gene names using predefined keyword lists. For each target gene, promoter regions were defined as 500 bp upstream of the gene start site, with appropriate strand orientation considered for reverse complement sequences. The Kobayashi motif pattern GT[ATCG]CGG[TG] was searched within these promoter sequences using regular expression matching with case-insensitive parameters. Motif occurrences were recorded with their genomic positions, sequences, and associated gene information for comparative analysis between the two strains.

### Statistical treatments

All statistical analyses were carried out in python language (*97*) using scipy.stats basic package (*98*) and statsmodel for advanced analysis of variance (ANOVA) designs (*99*). Groups of strains*temperatures were checked for normality with the Shapiro-Wilk test and for homogeneity of variances with Levene’s test. Within-culture temperature effects (20°C to 48 hours at 40°C) were analyzed with a repeated-measures ANOVA; when significant, post hoc pairwise comparisons used paired *t* tests. Between-strain differences at each temperature were tested with a one-way ANOVA (Welch’s ANOVA when homogeneity of variance was violated). If normality was violated, Wilcoxon signed-rank (within-culture) or Mann-Whitney *U* (between-strain) tests were used. For repeated-measures factors with >2 levels, if Mauchly’s test indicated sphericity violation, Greenhouse-Geisser correction was applied; for nonparametric repeated-measures scenarios, Friedman’s test with Dunn’s post hoc was used. Statistical significance was set at α = 0.05.
