# Supplementary material for: Compensatory respiration supports survival during extended heat stress in Microcystis aeruginosa
Source: Sci Adv. 2026 Jun 3;12(23):eadz4338. doi: 10.1126/sciadv.adz4338 (PMC13232611; doi:10.1126/sciadv.adz4338)
Supplement: Supplementary file 1 — Figs. S1 to S6 Tables S1 to S23 [file sciadv.adz4338_sm.pdf]

Science Advances
AAAS

Supplementary Materials for

Compensatory respiration supports survival during extended heat stress in *Microcystis aeruginosa*

Oded Liran *et al.*

Corresponding author: Oded Liran, oded.liran@ocean.org.il

Sci. Adv. **12**, eadz4338 (2026)
DOI: 10.1126/sciadv.adz4338

This PDF file includes:

Figs. S1 to S6
Tables S1 to S23

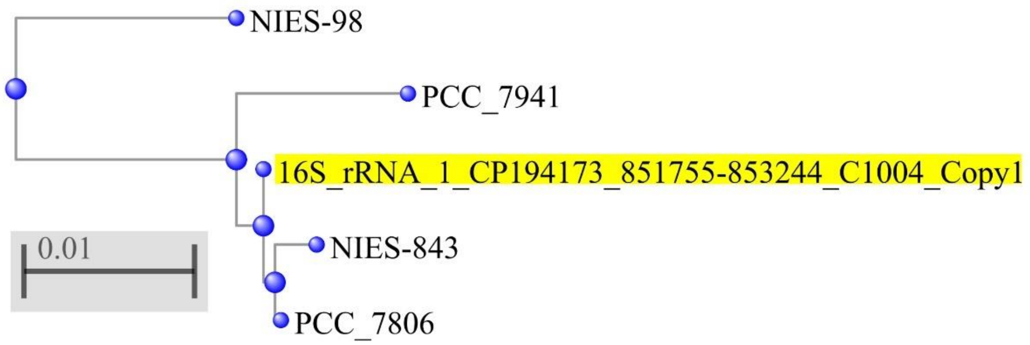

Figure S1. Distance tree between four *M. aeruginosa* strains' 16S rRNA sequences. The following accession numbers were used for the nodes in the figure: PCC7806 - CP155078.1, NIES-843 - NR_074314.1, PCC7941 - U40340.2, NIES 88 - U40337.2, C1004, sequenced genome.

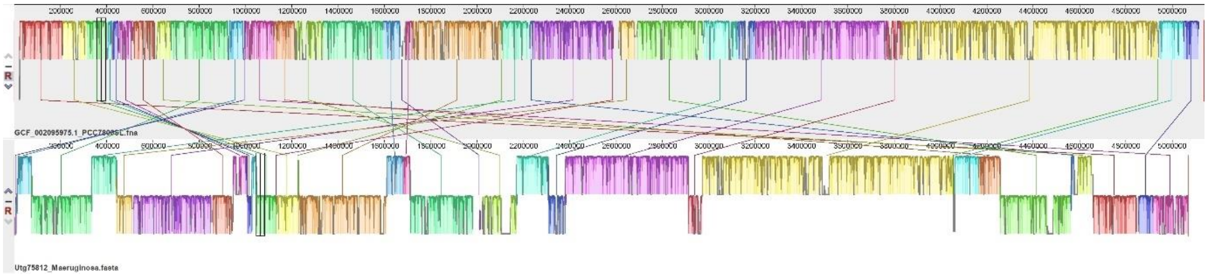

Figure S2. Whole-genome alignment of *M. aeruginosa* strains PCC7806 and C-1004 using Mauve. Mauve progressive alignment of the complete genomes of PCC7806 (top) and C-1004 (bottom). Coloured blocks represent locally collinear blocks (LCBs) of conserved sequence and gene order shared between the two genomes.

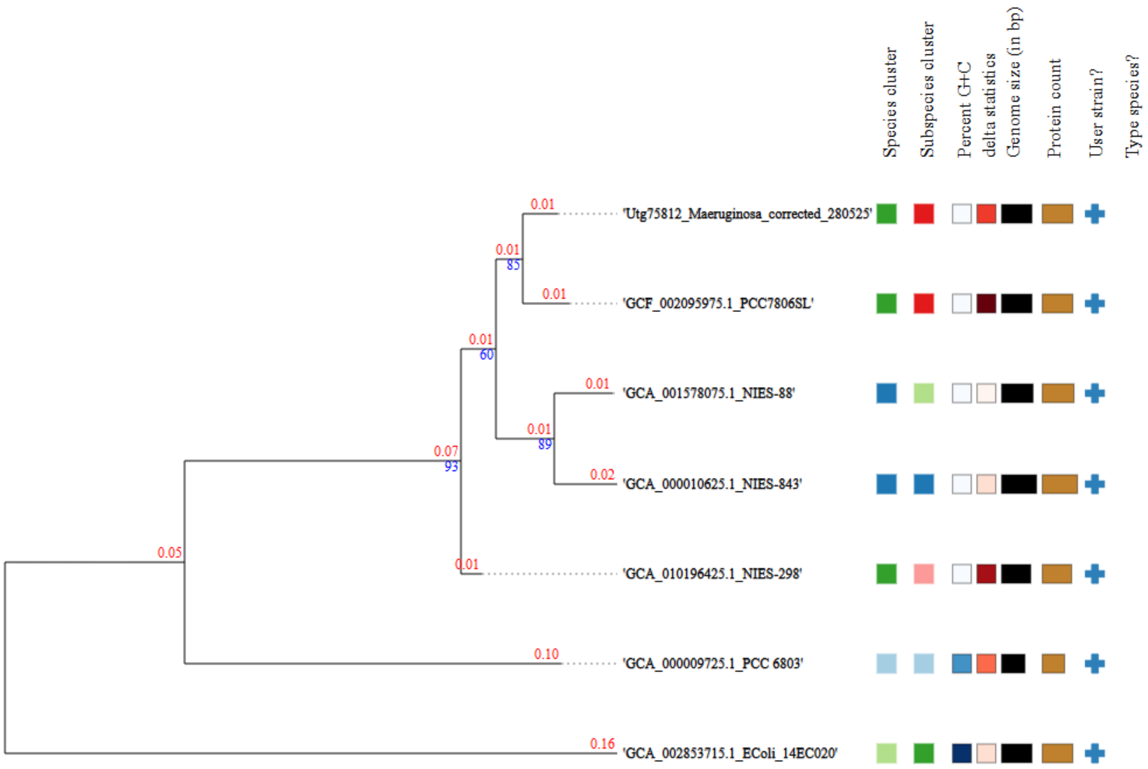

Figure S3. Genome BLAST Distance Phylogeny (GBDP) tree of *M. aeruginosa* strains PCC7806 and C-1004 and related genomes inferred by TYGS. Tree inferred with FastME 2.1.6.1 (*94*) from GBDP distances calculated from genome sequences. The branch lengths are scaled in terms of GBDP distance formula d5. The numbers above branches are GBDP pseudo-bootstrap support values > 60 % from 100 replications, with an average branch support of 81.8 %. The tree was rooted at the midpoint (*95*).

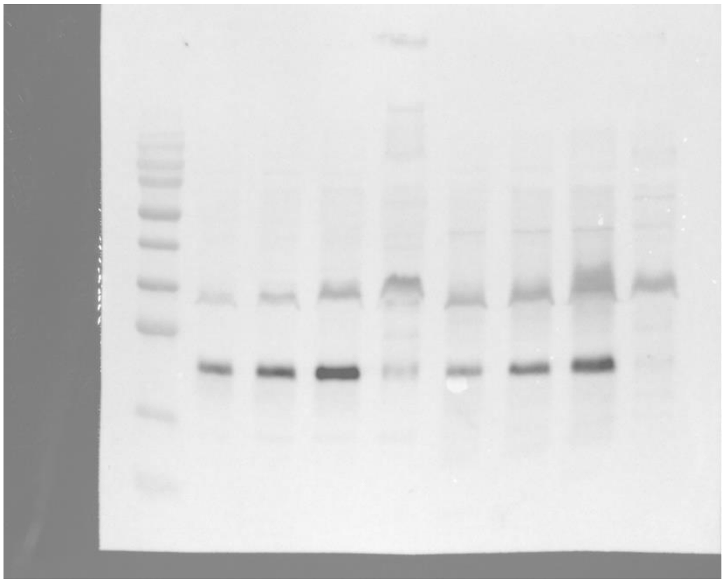

Figure S4. Figure 7 original PsbA figure.

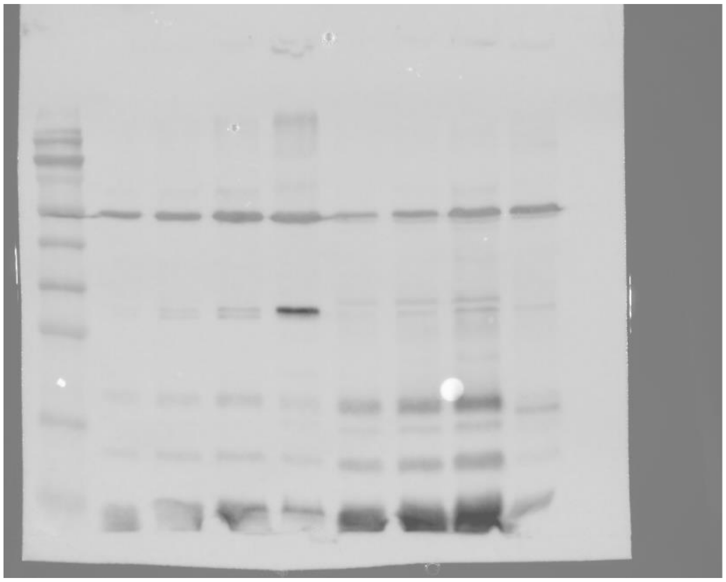

Figure S5. Figure 7 original PsaA figure.

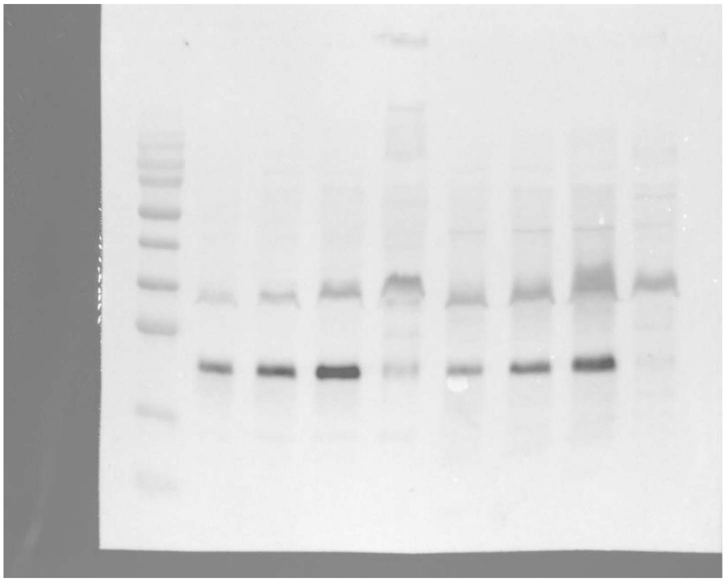

Figure S6. Figure 7 original cytB6 figure.

Table S1. Photosynthetic parameters related to the response of two *M. aeruginosa* strains to acute temperature stress. Data extracted from a statistical fit on a light response curve (*79*). The parameters checked: Light Use Efficiency (a.u.); Characteristic intensity (µmol photons $m^{-2}s^{-1}$); Optimum intensity (µmol photons $m^{-2}s^{-1}$); Maximum activity (a.u.).

Parameter	PCC7806		C-1004	
Temperature	20° C	40° C	20° C	40° C
Light Use Efficiency (Slope)	0.1 ± 0.01	0.02 ± 0.01	0.12 ± 0.01	0.03 ± 0.01
Optimum Intensity (Im)	1237.6 ± 290.4	1104.5 ± 391.4	1456.3 ± 346.4	1480.7 ± 533.2
Characteristic Intensity (Ik)	576.9 ± 122.3	551.2 ± 195.2	650.3 ± 194.5	721.0 ± 250.1
Maximum Photosynthesis (Pm)	51.7 ± 7.9[Ŧ]	5.5 ± 0.9[Ŧ]	74.8 ± 18.1[Ŧ]	24.8 ± 13.2[Ŧ]

[Ŧ]Statistical significant difference at $p<0.05$ for each strain between temperatures.

Table S2. Normalized differential expression in response to heat shock in two closely related *M. aeruginosa* strains. Normalized expression was quantified as the relative transcripts abundance of both strains in each temperature to a reference transcript PhosphoEnolPyruvate Carboxilase (PEPC) as suggested before (*87*). Values represent average of three biological repeats (each average on three technical repeats) and error equals standard error of the mean. Gene names refer to the following protein complexes: *groEL* – Chaperonin 60; *groES* – Co-Chapernonin 10; *clpB1* – HSP100 disaggregase; *dnaK* – Hsp70 Chaperone; *psbA* – D1 of PSII; *petE* – Plastocyanin; *petJ* – Cytochrome c_6; *sod* – SuperOxide Dismutase; *sdh* – Succinate DeHydrogenase; *icdh* – IsoCytrate DeHydrogenase.

Group	Gene	Species	T (° C)	Normalized Expression ($2^{-\Delta CT}$)
Heat shock	*groEL*	PCC7806	20	1.2 ± 0.23
			40	1.14 ± 0.09[^]
		C-1004	20	0.55 ± 0.01[***]
			40	0.87 ± 0.01[***,^]
	groES	PCC7806	20	20.04 ± 0.41[**,^]
			40	16.91 ± 0.66[**]
		C-1004	20	6.11 ± 0.91[**,^]
			40	17.00 ± 3.49[**]
	clpB1	PCC7806	20	0.08 ± 0.02
			40	0.1 ± 0.01[^]
		C-1004	20	0.08 ± 0.00[**]
			40	0.18 ± 0.02[**,^]
	dnaK	PCC7806	20	1.46 ± 0.04[**]
			40	1.95 ± 0.13[**]
		C-1004	20	1.47 ± 0.16
			40	2.18 ± 0.28
	psbA	PCC7806	20	158.68 ± 58.47

			40	102.68 ± 30.81
		C-1004	20	144.68 ± 56.00
			40	1025.33 ± 532.50
Photosynthesis related	*petE*	PCC7806	20	15.88 ± 2.04
			40	4.60 ± 0.38
		C-1004	20	4.55 ± 0.61
			40	4.21 ± 0.29
	petJ	PCC7806	20	2.10 ± 0.15$^{***,\wedge}$
			40	7.80 ± 0.47$^{***,\wedge}$
		C-1004	20	0.43 ± 0.03$^{***,\wedge}$
			40	2.49 ± 0.04$^{***,\wedge}$
	sod	PCC7806	20	0.13 ± 0.01***
			40	0.06 ± 0.00***
		C-1004	20	0.13 ± 0.01
			40	0.15 ± 0.05
TCA cycle related	*sdh*	PCC7806	20	0.22 ± 0.06$^{**,\wedge}$
			40	0.02 ± 0.01**
		C-1004	20	0.01 ± 0.01^
			40	5.79 ± 4.90
	icdh	PCC7806	20	0.78 ± 0.22$^{**,\wedge}$
			40	0.67 ± 0.05**
		C-1004	20	1.11 ± 0.04$^{\wedge}$
			40	2.08 ± 0.42

Table S3. Genome content comparison between two *M. aeruginosa* closely related strains.

Genome	ID	Length (bp)	GC (%)	AT (%)	A (%)	T (%)	G (%)	C (%)	Molecular Weight (Da)
C1004	CP194173	5,069,526	42.3	57.7	28.89	28.82	21.13	21.17	1,565,969,804
PCC7806SL	NZ_CP020771.1	5,139,339	42.09	57.91	28.94	28.97	21.05	21.03	1,587,596,944

Table S4. Genome sequence comparison between two closely related *M. aeruginosa* strains.

Metric	C1004_vs_PCC7806SL
Size Similarity	98.64%
Composition Similarity	99.90%
K-mer Jaccard Similarity	100.00%
K-mer Cosine Similarity	99.92%
Direct Sequence Similarity	25.75%
Overall Similarity	77.52%

Table S5. Genome-based similarity metrics. Genome-based similarity metrics (dDDH and G+C content) derived from TYGS analysis of the *Microcystis aeruginosa* C-1004 relative to reference strain *Microcystis aeruginosa* PCC-7806**.**

Query strain	Subject strain	dDDH (d0, in %)	C.I. (d0, in %)	dDDH (d4, in %)	C.I. (d4, in %)	dDDH (d6, in %)	C.I. (d6, in %)	G+C content difference (in %)
'GCF 002095975.1 PCC7806SL'	'Utg75812 Maeruginosa corrected 280525'	87.1	[83.6 - 90.0]	81.7	[78.8 - 84.3]	89.1	[86.2 - 91.4]	0.21
'GCA 010196425.1 NIES-298'	'Utg75812 Maeruginosa corrected 280525'	77.6	[73.6 - 81.1]	75.1	[72.1 - 77.9]	80	[76.6 - 83.0]	0.26
'GCA 000010625.1 NIES-843'	'GCA 001578075.1 NIES-88'	71.5	[67.5 - 75.1]	75	[72.0 - 77.7]	74.6	[71.1 - 77.8]	0.4
'GCA 010196425.1 NIES-298'	'GCF 002095975.1 PCC7806SL'	78.9	[74.9 - 82.4]	71.5	[68.5 - 74.3]	80.4	[77.0 - 83.4]	0.47
'GCA 001578075.1 NIES-88'	'GCA 010196425.1 NIES-298'	62.9	[59.1 - 66.5]	65.2	[62.2 - 68.0]	64.9	[61.6 - 68.1]	0.18
'GCA 000010625.1 NIES-843'	'GCA 010196425.1 NIES-298'	61.2	[57.5 - 64.8]	65	[62.1 - 67.9]	63.4	[60.1 - 66.6]	0.22
'GCA 000010625.1 NIES-843'	'Utg75812 Maeruginosa corrected 280525'	61.1	[57.4 - 64.7]	64.4	[61.4 - 67.2]	63.2	[59.8 - 66.4]	0.03
'GCA 001578075.1 NIES-88'	'Utg75812 Maeruginosa corrected 280525'	58.8	[55.2 - 62.3]	63.9	[61.0 - 66.7]	60.9	[57.7 - 64.1]	0.43
'GCA 000010625.1 NIES-843'	'GCF 002095975.1 PCC7806SL'	59.8	[56.1 - 63.3]	63.4	[60.5 - 66.2]	61.8	[58.5 - 64.9]	0.24

'GCA 001578075.1 NIES-88'	'GCF 002095975.1 PCC7806SL'	60.2	[56.5 - 63.7]	63	[60.1 - 65.8]	62	[58.7 - 65.2]	0.64
'GCA 000009725.1 PCC 6803'	'GCA 010196425.1 NIES-298'	12.6	[9.9 - 15.8]	27.4	[25.0 - 29.8]	13	[10.7 - 15.7]	4.81
'GCA 000009725.1 PCC 6803'	'GCF 002095975.1 PCC7806SL'	12.6	[9.9 - 15.8]	25.1	[22.8 - 27.6]	13	[10.7 - 15.7]	5.28
'GCA 000009725.1 PCC 6803'	'GCA 001578075.1 NIES-88'	12.6	[9.9 - 15.8]	24.1	[21.8 - 26.6]	13	[10.7 - 15.7]	4.64
'GCA 000009725.1 PCC 6803'	'Utg75812 Maeruginosa corrected 280525'	12.6	[9.9 - 15.9]	23.7	[21.4 - 26.2]	13	[10.7 - 15.7]	5.07
'GCA 000009725.1 PCC 6803'	'GCA 000010625.1 NIES-843'	12.6	[9.9 - 15.9]	22.4	[20.2 - 24.9]	13	[10.7 - 15.7]	5.04
'GCA 002853715.1 EColi 14EC020'	'GCA 010196425.1 NIES-298'	12.6	[9.9 - 15.8]	16.5	[14.4 - 18.8]	13	[10.6 - 15.7]	8.15
'GCA 002853715.1 EColi 14EC020'	'GCF 002095975.1 PCC7806SL'	12.6	[9.9 - 15.8]	16.1	[14.0 - 18.4]	13	[10.6 - 15.7]	8.61
'GCA 000009725.1 PCC 6803'	'GCA 002853715.1 EColi 14EC020'	12.6	[9.9 - 15.8]	16.1	[14.0 - 18.4]	13	[10.6 - 15.7]	3.33
'GCA 002853715.1 EColi 14EC020'	'Utg75812 Maeruginosa corrected 280525'	12.6	[9.9 - 15.8]	15.9	[13.8 - 18.2]	13	[10.6 - 15.7]	8.4
'GCA 001578075.1 NIES-88'	'GCA 002853715.1 EColi 14EC020'	12.5	[9.9 - 15.8]	15.9	[13.8 - 18.2]	12.9	[10.6 - 15.7]	7.97

'GCA 000010625.1 NIES-843'	'GCA 002853715.1 EColi 14EC020'	12.6	[9.9 - 15.8]	15.9	[13.8 - 18.1]	12.9	[10.6 - 15.7]	8.37

Table S6. Hik34-Rre1 Canonical motif appearance on promoters of pertinent genes in two *M. aeruginosa* strains.

**genome**	**Pathway**	**product**	**Motif sequence**	**Motif position**
C1004	Terminal Oxidases	cytochrome c oxidase subunit 3	GTTCGGT	199
C1004	Heat Shock	ATP-dependent chaperone ClpB	GTACGGT	334
C1004	Heat Shock	RuBisCO chaperone RbcX	GTTCGGT	144
C1004	Heat Shock	co-chaperone GroES	GTTCGGG	371
C1004	Heat Shock	chaperonin GroEL	GTTCGGG	12
C1004	Heat Shock	DnaJ C-terminal domain-containing protein	GTACGGG	98
C1004	Heat Shock	Hsp70 family protein	GTACGGT	2
C1004	Heat Shock	Hsp20/alpha crystallin family protein	GTACGGG	375
C1004	TCA Cycle	citrate synthase	GTTCGGT	69
C1004	TCA Cycle	NADP-dependent isocitrate dehydrogenase	GTTCGGT	297
C1004	TCA Cycle	succinate dehydrogenase/fumarate reductase iron-sulfur subunit	GTTCGGT, GTGCGGG	92, 198
C1004	Light Reactions	photosystem I reaction center subunit IV	GTTCGGG	378
C1004	Light Reactions	photosystem II S4 domain protein	GTTCGGT, GTGCGGG	46, 313
C1004	Light Reactions	ferredoxin--nitrite reductase	GTACGGG	150
C1004	Light Reactions	phosphoenolpyruvate synthase	GTCCGGG	8
C1004	Light Reactions	sulfite reductase, ferredoxin dependent	GTACGGT	335
C1004	Light Reactions	ferredoxin	GTCCGGT	363
C1004	Light Reactions	photosystem II reaction center protein PsbX	GTGCGGT	423
C1004	Light Reactions	cytochrome f	GTACGGG	59
C1004	Light Reactions	photosystem I reaction center subunit PsaK	GTCCGGT, GTGCGGG	77, 456
C1004	Light Reactions	ferredoxin:protochlorophyllide reductase (ATP-dependent) iron-sulfur ATP-binding protein	GTCCGGG	122
C1004	Light Reactions	photosystem II protein Y	GTCCGGG	192
C1004	Light Reactions	photosystem II q(b) protein	GTTCGGG	149
C1004	Light Reactions	photosystem I iron-sulfur center protein PsaC	GTTCGGG	305

C1004	Light Reactions	photosystem I reaction center subunit II PsaD	GTTCGGT	103
C1004	Light Reactions	photosystem II reaction center protein K	GTGCGGG	168
C1004	Light Reactions	protochlorophyllide reductase	GTACGGG	62
C1004	Light Reactions	(2Fe-2S) ferredoxin domain-containing protein	GTCCGGG	240
PCC7806	Terminal Oxidases	Cytochrome c oxidase subunit 1	GTACGGT	234
PCC7806	Heat Shock	Chaperone protein ClpB	GTTCGGG	81
PCC7806	Heat Shock	Chaperone protein dnaK2	GTTCGGT, GTTCGGG	2, 485
PCC7806	Heat Shock	Chaperone protein dnaK2	GTTCGGT	386
PCC7806	Heat Shock	Chaperone protein DnaK	GTACGGG	440
PCC7806	Light Reactions	Light-independent protochlorophyllide reductase subunit B	GTTCGGG	316
PCC7806	Light Reactions	Ferredoxin-dependent glutamate synthase 1	GTACGGT	148
PCC7806	Light Reactions	Light-independent protochlorophyllide reductase subunit B	GTACGGT	417
PCC7806	Light Reactions	Alkene monooxygenase system%2C ferredoxin component	GTACGGT	224
PCC7806	Light Reactions	Glutamate synthase [NADPH] small chain	GTACGGG	29
PCC7806	Light Reactions	Photosystem II reaction center protein I	GTACGGG	250
PCC7806	Light Reactions	Photosystem II reaction center protein J	GTGCGGG	385
PCC7806	Light Reactions	Iron stress-induced chlorophyll-binding protein	GTTCGGT	254
PCC7806	Light Reactions	Photosystem II D2 protein	GTTCGGT	380
PCC7806	Light Reactions	Photosystem I reaction center subunit PsaK 2	GTTCGGT	383
PCC7806	Light Reactions	Photosystem II CP43 reaction center protein	GTACGGG	82
PCC7806	Light Reactions	Photosystem II D2 protein	GTGCGGG	69
PCC7806	Light Reactions	Cytochrome b6	GTACGGT	486
PCC7806	Light Reactions	Photosystem I assembly protein Ycf3	GTTCGGG	159
PCC7806	Light Reactions	30S ribosomal protein S1	GTTCGGG	93
PCC7806	Light Reactions	NADPH-dependent quinone reductase ArsH	GTTCGGT	95
PCC7806	Light Reactions	NADPH-dependent quinone reductase ArsH	GTACGGG	415
PCC7806	Light Reactions	Sulfite reductase [ferredoxin]	GTTCGGG, GTCCGGT	266, 461
PCC7806	Light Reactions	Photosystem I assembly protein Ycf3	GTTCGGT	138
PCC7806	Light Reactions	Ferredoxin-1	GTTCGGT	352, 376
PCC7806	Light Reactions	Ferredoxin--NADP reductase	GTACGGG	112
PCC7806	Light Reactions	Photosystem I reaction center subunit VIII	GTCCGGG	301

PCC7806	Light Reactions	Photosystem I reaction center subunit XI	GTCCGGG	475
PCC7806	Light Reactions	Ferredoxin-thioredoxin reductase%2C variable chain	GTGCGGT	36
PCC7806	Light Reactions	Ferredoxin-dependent glutamate synthase 2	GTGCGGG	87

Table S7. List of primers used to analyze expression levels of respiration and photosynthesis related genes. Genes included: Cytochrome C_6 (PetJ), Plastocyanin (PetE), D1 subunit of PSII (Psba), Succinate Dehydrogenase (Sdha), Isocitrate dehydrogenase (NADP+) (ICDP), Super oxide dismutase (SOD), PhosphoEnol Pyruvate Carboxylase (PPC). Primers were designed based on the PCC7806SL genome.

Name	Forward sequence	Reverse sequence
petJ	TGGGGCTTCGATTTTCAGTGCTAAC	AACGATAGCTTCAACGGAATCTTTGCC
petE	GTTATTGACCCATTTCACCGTATCTCC	TCTCTAGCTTCTTCTTCAACACTGCC
psba	ATGATCCCCACCCTGCTCACCGCCACC	GGAGATGATGTTGTTTCCGTAGAGTAG
sdha	TCCTCGGTTCCCTCGGAAGCTTCAAAG	GGGCAATTATTGGCGCGATTTACAGGG
icdp	TCGCCAATATTCGCCCCGGGTCCCATC	TATCTTCCAACAGATCCAAACCCGTCC
sod	CTCCCAGTTAACCACATTCCACCACTG	ATTATCAGCACTGCTAACCAAGATAGC
ppc	TCT GTT CTG AGG AAG GAC AAG TGA CCG	ACT GGA AAT AGA GGG CAA AAG CGC GGG
groEL	CCACAGAAACCGAACTCAAGGATC	AGAAGCCAGATGAATCAAGGTTGTAC
groES	ACAACTGTTAAACCCCTAGGCGATCG	GGTCACGACTTCCCCGATTTGG
clpB1	AGAGCAGAATAACGGTCTAGCCACC	AGCAGCAGATCGAGACCACGACCTAGG
dnaK1	TCGAATACCTTCACCAATAGGCCAACC	CTCATAGTCACTGGTGCTGATTTCTTC

Table S8. Figure 2 Data

Strain	Temperature	Values
PCC7806	20	0.039381
PCC7806	20	0.036965
PCC7806	20	0.047469
PCC7806	20	0.046988
PCC7806	24	0.081358
PCC7806	24	0.108347
PCC7806	24	0.087637
PCC7806	24	0.081841
PCC7806	32	0.122251
PCC7806	32	0.150592
PCC7806	32	0.215041
PCC7806	32	0.103814
C1004	20	0.075465
C1004	20	0.070436
C1004	20	0.102193
C1004	20	0.086015
C1004	24	0.089175
C1004	24	0.1125
C1004	24	0.094225
C1004	24	0.11395
C1004	32	0.151104
C1004	32	0.199996
C1004	32	0.130362
C1004	32	0.145968

Table S9. Figure 3 Data

**Species**	**Temperature**	**Value**
C-1004	20	49300000
C-1004	20	57400000
C-1004	20	65500000
C-1004	40	34810000
C-1004	40	35800000
C-1004	40	35305000
PCC7806	20	70000000
PCC7806	20	53800000
PCC7806	20	58300000
PCC7806	40	55510000
PCC7806	40	54700000
PCC7806	40	55105000

Table S10. Figure 4A Data

**7806**	**I**	**II**	**III**
**0**	0	0	0
**20**	1.763172	2.036015	2.152409
**40**	3.354076	4.496395	4.577784
**67**	5.248817	7.159388	7.320329
**184**	12.41847	15.36555	18.96544
**320**	19.46611	29.35388	29.43719
**640**	35.31798	45.0329	48.5095
**1200**	49.64267	36.47828	59.14275
**2400**	42.56729	10.25162	62.0485
**C-1004**	**I**	**II**	**III**
**0**	0	0	0
**20**	2.766411	2.531839	2.211218
**40**	5.616817	5.270962	4.733003
**67**	8.757722	8.46664	7.220345
**184**	24.34062	20.80689	19.78872
**320**	36.2104	32.51933	29.71446
**640**	62.94706	55.54983	42.92175
**1200**	88.05445	70.98119	33.51004
**2400**	88.65783	83.18351	27.01751

Table S11. Figure 4A Model

PCC7806	I	II	III	Average	C1004	I	II	III	Average
0	0	0	0	0	0	0	0	0	0
10	0.685856718	1.189711315	0.972571	0.94938	10	1.182277617	1.019863	1.456137	1.219426
20	1.371430719	2.377814168	1.940606	1.896617	20	2.364288436	2.037128	2.874874	2.42543
30	2.056522509	3.562707336	2.903916	2.841049	30	3.545765862	3.051645	4.256315	3.617909
40	2.740933079	4.74280401	3.862314	3.782017	40	4.726443699	4.063266	5.600628	4.796779
50	3.424464098	5.916538831	4.815617	4.718873	50	5.906056351	5.071845	6.908042	5.961981
60	4.106918098	7.082374723	5.763649	5.650981	60	7.084339025	6.077235	8.178836	7.11347
70	4.78809867	8.238809471	6.706234	6.577714	70	8.26102792	7.079295	9.413342	8.251222
80	5.467810647	9.384381971	7.643203	7.498465	80	9.435860432	8.077883	10.61194	9.375226

90	6.145860292	10.51767811	8.574391	8.412643	90	10.60857534	9.072859	11.77503	10.48549
100	6.822055473	11.63733623	9.499638	9.319676	100	11.77891301	10.06409	12.90309	11.58203
200	13.44123906	21.83022037	18.39319	17.88822	200	23.29591948	19.74158	22.3766	21.8047
300	19.68188705	29.67580773	26.55007	25.30259	300	34.30817153	28.91009	29.00284	30.74037
400	25.40179351	34.97747618	33.88634	31.42187	400	44.60809854	37.47211	33.42304	38.50108
500	30.50079243	38.04691388	40.36207	36.30326	500	54.03464853	45.3567	36.20606	45.19914
600	34.92284215	39.4050804	45.9752	40.10104	600	62.4785654	52.51934	37.80726	50.93505
700	38.65253681	39.56968772	50.75367	42.99197	700	69.88192615	58.93987	38.57138	55.79773
800	41.70789982	38.9612767	54.74713	45.13877	800	76.23309349	64.61946	38.75135	59.86797
900	44.13151533	37.88650492	58.01917	46.67906	900	81.55871266	69.5768	38.52939	63.22164
1000	45.98170794	36.55483021	60.64085	47.7258	1000	85.91440664	73.84416	38.03552	65.93136
1100	47.32488327	35.10314378	62.68558	48.3712	1100	89.37553319	77.46352	37.36196	68.06701
1200	48.22954923	33.61783676	64.22538	48.69092	1200	92.02893298	80.4832	36.57391	69.69535
1300	48.76208706	32.15155605	65.32843	48.74736	1300	93.96616276	82.95488	35.71731	70.87945
1400	48.98407136	30.73496957	66.05754	48.59219	1400	95.27835615	84.93131	34.82445	71.67804
1500	48.95081963	29.3846894	66.46938	48.2683	1500	96.05261809	86.4645	33.91791	72.14501
1600	48.71083669	28.10848352	66.61432	47.81121	1600	96.36972865	87.60439	33.01332	72.32914
1700	48.30585887	26.90865687	66.53651	47.25034	1700	96.30288415	88.398	32.1214	72.27409
1800	47.77126483	25.78422507	66.2743	46.60993	1800	95.91720811	88.88891	31.24933	72.01848
1900	47.13668234	24.73230197	65.86072	45.9099	1900	95.26979841	89.11695	30.40179	71.59618
2000	46.42667438	23.74897858	65.32402	45.16656	2000	94.41012157	89.11816	29.58165	71.03664
2100	45.66143027	22.82987398	64.68822	44.39318	2100	93.38061039	88.92485	28.7905	70.36532
2200	44.85741798	21.97047538	63.97367	43.60052	2200	92.2173614	88.5658	28.02904	69.60407
2300	44.02797488	21.16634269	63.19753	42.79728	2300	90.95086072	88.06644	27.2973	68.77154

2400	43.18382792	20.41322647	62.37419	41.99041	2400	89.6066927	87.44917	26.5949	67.88359

Table S12. Figure 4B Data

PCC7806	I	II	III
0	0	0	0
20	0.24027	0.417613	0.94843
40	0.33304	0.578855	1.558735
67	0.605764	1.052876	1.470499
184	1.320962	2.295958	4.229594
320	1.728176	3.003734	3.978907
640	1.658535	2.882691	6.706545
1200	5.868547	10.20009	0
2400	3.127631	5.43612	0
C-1004	I	II	III
0	0	0	0
20	0.695564	0.668512	0.696909
40	0.780356	1.823684	1.853449
67	1.016796	1.770714	2.85016
184	1.598383	2.160789	8.602059
320	4.998442	6.068227	11.01841
640	6.823121	16.0932	20.6918
1200	4.099021	5.538462	24.122
2400	0.097373	16.85619	53.45816

Table S13. Figure 4B Model

**PCC7806**	**I**	**II**	**III**	**Average**	**C1004**	**I**	**II**	**III**	**Average**
**0**	0	0	0	0	0	0	0	0	0
**10**	0.057523082	0.099891755	0.297674	0.151696	10	0.191736	5.8808E-05	0.437847	0.209881
**20**	0.115024127	0.199577353	0.593628	0.302743	20	0.383095	5.8811E-05	0.875645	0.4196
**30**	0.172487739	0.299030788	0.886175	0.452564	30	0.573705	5.8811E-05	1.313347	0.629037
**40**	0.229898554	0.398226287	1.173692	0.600606	40	0.763198	5.881E-05	1.750909	0.838055
**50**	0.287241257	0.497138333	1.454652	0.746344	50	0.951212	5.8809E-05	2.188285	1.046519
**60**	0.344500598	0.595741692	1.727644	0.889295	60	1.137397	5.8808E-05	2.62543	1.254295

**70**	0.401661397	0.694011424	1.991399	1.029024	70	1.321412	5.8807E-05	3.062297	1.461256
**80**	0.458708567	0.791922911	2.244804	1.165145	80	1.50293	5.8806E-05	3.498843	1.667277
**90**	0.51562712	0.889451872	2.486915	1.297331	90	1.681642	5.8805E-05	3.935021	1.872241
**100**	0.572402185	0.986574385	2.716957	1.425311	100	1.857253	5.8804E-05	4.370787	2.076033
**200**	1.129095832	1.930471258	4.297429	2.452332	200	3.392369	5.8793E-05	8.695982	4.02947
**300**	1.656386717	2.810664188	4.779957	3.082336	300	4.445982	5.8781E-05	12.93194	5.79266
**400**	2.142990349	3.611252161	4.6798	3.478014	400	5.037442	5.8769E-05	17.038	7.358501
**500**	2.580689129	4.321993793	4.360169	3.754284	500	5.277488	5.8757E-05	20.97775	8.751764
**600**	2.964543595	4.938122524	3.990311	3.964326	600	5.286967	5.8745E-05	24.71991	10.00231
**700**	3.292706661	5.459627964	3.635551	4.129295	700	5.160754	5.8733E-05	28.23903	11.13328
**800**	3.565955691	5.890238181	3.3166	4.257598	800	4.962794	5.8721E-05	31.51574	12.15953
**900**	3.787078523	6.236319658	3.036767	4.353388	900	4.732661	5.8709E-05	34.53675	13.08982
**1000**	3.960234061	6.505854433	2.793189	4.419759	1000	4.493491	5.8697E-05	37.29458	13.92938
**1100**	4.090372793	6.70758644	2.581258	4.459739	1100	4.258147	5.8685E-05	39.78703	14.68174
**1200**	4.18276368	6.850371076	2.396296	4.476477	1200	4.033337	5.8673E-05	42.01657	15.34999
**1300**	4.242641695	6.942721855	2.234115	4.473159	1300	3.822175	5.8662E-05	43.98964	15.93729
**1400**	4.274968777	6.992525756	2.09115	4.452882	1400	3.625737	5.865E-05	45.71582	16.44721
**1500**	4.284289759	7.006890582	1.964433	4.418538	1500	3.443967	5.8638E-05	47.20718	16.88374
**1600**	4.27466114	6.992088079	1.85151	4.372753	1600	3.276218	5.8626E-05	48.47753	17.25127
**1700**	4.249631649	6.953561634	1.75036	4.317851	1700	3.121558	5.8614E-05	49.54182	17.55448
**1800**	4.212256892	6.895973992	1.659312	4.255848	1800	2.978952	5.8602E-05	50.41565	17.79822
**1900**	4.165134385	6.823276936	1.57698	4.188464	1900	2.847351	5.859E-05	51.11476	17.98739
**2000**	4.11044907	6.738790405	1.502211	4.11715	2000	2.725752	5.8578E-05	51.65474	18.12685
**2100**	4.0500226	6.645282904	1.434038	4.043114	2100	2.613221	5.8567E-05	52.05071	18.22133

**2200**	3.985362089	6.545048244	1.371647	3.967353	2200	2.508903	5.8555E-05	52.31713	18.27537
**2300**	3.917705813	6.439975884	1.31435	3.890677	2300	2.412023	5.8543E-05	52.4677	18.29326
**2400**	3.848064581	6.331613686	1.26156	3.813746	2400	2.321883	5.8531E-05	52.5152	18.27905

Table S14. Figure 5A Data

C-1004	20C					PCC7806	20C			
move to Zero						move to Zero				
Excitation 435						Excitation 435				
	I	II	III	Average			I	II	III	Average
670	33053.4	3600.2	188225.3	74959.63		670	229795.1	8724.63	75034.1	104517.9
671	0	0	173777.1	57925.7		671	203923.8	6687.69	67309.4	92640.3
672	16198.6	9679.5	180828.8	68902.3		672	201295.4	6716.05	57145.2	88385.55
673	20146.6	16021.5	177596	71254.7		673	173418.8	5720.31	51605.3	76914.8
674	31015.3	35379.4	161010.1	75801.6		674	168192	5838.93	51422.6	75151.18
675	61761.4	54103.4	181613.8	99159.53		675	149668.8	5028.69	57182.2	70626.56
676	111089.8	79222.4	211968.4	134093.5		676	171921.3	5324.39	55087.8	77444.5
677	157760.2	107725.7	232606.5	166030.8		677	169341.3	5337.74	48511.8	74396.95
678	242683.9	144894	260773.1	216117		678	197357.9	6193.13	50749.5	84766.84
679	332097.7	174164.7	270203.5	258822		679	217845.1	7185.6	56954.2	93994.97
680	414846	212369.2	295191.5	307468.9		680	227347.1	6737.79	70898.8	101661.2
681	494579	297165.3	291341.7	361028.7		681	217361	6726.07	74736.7	99607.92
682	501684	288770.2	269000.4	353151.5		682	234117	6523.93	75246.7	105295.9
683	492742	274765.3	243534.2	337013.8		683	210104.9	6301.7	66781.4	94396
684	439862	230713.3	204473.8	291683		684	217355.2	5473.05	61394.8	94741.02
685	414182	242235.8	190644.1	282354		685	192888.4	5382.85	57684.3	85318.52
686	377259.1	217615.5	152150.2	249008.3		686	157721.9	4950.17	52958	71876.69
687	349345.9	204849.9	148319.6	234171.8		687	124517.5	3846.03	48139.7	58834.41
688	317843.2	181924.7	144210	214659.3		688	129944.2	3443.5	45843.2	59743.63
689	358510.3	173610.2	142599.6	224906.7		689	99404.3	3375	34244.8	45674.7
690	310555.1	189188.2	110102.7	203282		690	113280.5	3099.42	25814.3	47398.07
691	318906.1	171741	82117.1	190921.4		691	93248.7	1840.16	20435.3	38508.05
692	275432.3	155693.4	98332.3	176486		692	97547.8	1284.08	20826.7	39886.19
693	264236.6	134866.4	79615.4	159572.8		693	74950.8	1694.87	18191.4	31612.36
694	216001.3	134878.3	45756.6	132212.1		694	58266.6	923.38	20506.2	26565.39
695	200846.8	124418.6	28525.3	117930.2		695	52811.3	681.29	14539.5	22677.36
696	149256.8	108629.6	17509.8	91798.73		696	39295.9	465.86	12711	17490.92
697	124401.3	89700.7	24163.3	79421.77		697	48223.3	1043.65	2556.4	17274.45
698	72696.9	55320	9313.5	45776.8		698	35908.6	561.03	5590	14019.88
699	58700.5	45076.9	12392.2	38723.2		699	34877.9	814.85	2504.9	12732.55
700	30877.3	45772.1	0	25549.8		700	39621.1	1546.25	2263.3	14476.88
701	28938.9	38334.7	21148.3	29473.97		701	34597.7	1405.98	0	12001.23
702	22354.6	52998.9	40645.9	38666.47		702	62036.8	2289.4	9326.4	24550.87
703	72621.3	76477.2	64335	71144.5		703	81030.8	2439.71	29506.8	37659.1

704	113292.1	100987.7	110624.4	108301.4		704	94205.6	4437.33	41163.5	46602.14
705	171423.6	127255.7	134535.5	144404.9		705	159966.8	4689.57	62059.6	75571.99
706	239552.1	167167.8	167395	191371.6		706	204024.1	6433.7	88591.3	99683.03
707	270010.8	234146.9	216928	240361.9		707	264023.3	8094.56	107517.4	126545.1
708	383113.6	285715.3	262130.2	310319.7		708	316928.3	9013.69	118168.9	148037
709	462483	326388.5	323937.6	370936.4		709	357291.1	10557.98	127789.1	165212.7
710	478364	389483.8	376114	414653.9		710	424352	12692.69	154499.4	197181.4
711	647625	450486.3	417268.5	505126.6		711	479927.3	14879.62	191642.7	228816.5
712	721986	528031	504205.5	584740.8		712	510722.9	16076.95	229749.1	252183
713	771954	588118.9	549317.6	636463.5		713	585655.3	17175.75	262592.1	288474.4
714	897469	595908.7	584697.7	692691.8		714	667525.4	19609.69	267965.4	318366.8
715	946073	712465.7	669490.4	776009.7		715	682925.6	21448.4	303640.5	336004.8
716	1015989	754914.9	710411.4	827105.1		716	749645	21696.09	309575.2	360305.4
717	1035389	731825.9	739025.4	835413.4		717	788424	23208.88	306500	372711
718	1061302	771855.9	721711.4	851623.1		718	801487	23727.73	328100.5	384438.4
719	1108122	782117.9	775004.4	888414.8		719	812099	23439.75	322300	385946.3
720	1175751	808379.9	792867.4	925666.1		720	839308	22792.1	325488.3	395862.8
721	1103347	758241.9	762113.4	874567.4		721	818899	23334.34	323633.2	388622.2
722	1115539	796223.9	741354.4	884372.4		722	788569	22618.09	320992.3	377393.1
723	1128799	777312.9	768458.4	891523.4		723	780240	22889.2	286182.8	363104
724	1077381	784766.9	714657.4	858935.1		724	758013	21286.13	278802.8	352700.6
725	1071547	800880.9	674110.4	848846.1		725	693522.5	21520.71	251239.9	322094.4
726	918828	758764.9	683139.4	786910.8		726	691945.3	20710.73	242445.9	318367.3
727	927708	713688.3	655056.5	765484.3		727	640504.6	19107.87	219797.7	293136.7
728	957905	633859.3	590552.7	727439		728	639117.9	17896.69	204282.8	287099.1
729	880968	643573.3	564087.6	696209.6		729	597404.4	16811.32	184922.3	266379.3
730	749020	581657.7	525942.1	618873.3		730	587304.4	16769.62	160372.5	254815.5
731	733429	510866.6	507245.4	583847		731	544107.7	14618.91	148540.2	235755.6
732	658565	497195.6	475091.3	543617.3		732	501613.1	13296.29	138616.6	217842
733	604440	427506.1	448187.7	493377.9		733	435198.3	11597.75	132753.2	193183.1
734	525786	422787.9	400054.2	449542.7		734	381823.8	10837.14	127402.2	173354.4
735	508878	390881.5	358993.7	419584.4		735	331849.3	9642.17	120507.1	153999.5
736	481629	359231.4	307282.9	382714.4		736	266822.4	8300.2	117329.3	130817.3
737	417201	309567	267551.2	331439.7		737	269404.3	7192.29	96726.1	124440.9
738	333881.7	277811.2	244370.7	285354.5		738	219597.5	6084.57	83597.2	103093.1
739	269075.3	222221.1	205304.1	232200.2		739	197168.1	5002.02	66360.7	89510.27
740	252046.4	213977.1	179588	215203.8		740	152369.7	4266.97	62898.9	73178.52

Table S15. Figure 5B data

C-1004	40C					PCC7806	40C			
move to Zero						move to Zero				
Excitation 435						Excitation 435				
	I	II		Average			I	II		Average
670	6734.1	0		3367.05		670	1288.45	3284.88		2286.665
671	5848.1	1172.36		3510.23		671	1251.68	2496.25		1873.965
672	11977.5	1804.14		6890.82		672	1126.35	2153.42		1639.885
673	15730.1	3688.31		9709.205		673	715.25	1570.32		1142.785
674	20455.7	5451.18		12953.44		674	0	1627.07		813.535
675	24104.8	6610.72		15357.76		675	1323.54	2166.75		1745.145
676	29544.8	7739.99		18642.4		676	2548.7	2111.64		2330.17
677	32633.6	8622.14		20627.87		677	1505.75	2445.74		1975.745
678	30798.8	10551.54		20675.17		678	3085.25	2926.99		3006.12
679	46133.3	12873.42		29503.36		679	2904.69	2429.08		2666.885
680	49468.7	15038.43		32253.57		680	3560.05	2659.57		3109.81
681	51676.2	16448.18		34062.19		681	3411.22	3217.71		3314.465
682	57094.9	18911.67		38003.29		682	4006.29	2883.47		3444.88
683	53340.9	17284.59		35312.75		683	3330.96	2053.06		2692.01
684	44544.4	17317.53		30930.97		684	2983.28	1705.56		2344.42
685	41742.8	15017.84		28380.32		685	2789.36	1469.97		2129.665
686	31549	12180.42		21864.71		686	1689.57	978.84		1334.205
687	25504.8	11545.05		18524.93		687	1370.34	372.43		871.385
688	19169.5	8735.65		13952.58		688	1071.19	0		535.595
689	11401.2	7228.94		9315.07		689	2087.34	826.92		1457.13
690	9075.4	6640.73		7858.065		690	1977.05	905.49		1441.27
691	9896.8	4968.47		7432.635		691	1520.76	402.73		961.745
692	9721.9	4750.78		7236.34		692	1288.46	757.03		1022.745
693	6757.8	4375.34		5566.57		693	945.86	1552.53		1249.195
694	0	3132.2		1566.1		694	1554.18	1664.76		1609.47
695	1226.3	2637.91		1932.105		695	1898.49	2386.87		2142.68
696	3344.1	3438.88		3391.49		696	1485.68	3029.11		2257.395
697	3804.5	2148.78		2976.64		697	1352	4114.08		2733.04
698	5370.9	3269.52		4320.21		698	1930.23	4269.21		3099.72
699	6441.1	3732.07		5086.585		699	2595.48	5244.13		3919.805
700	10800.2	4780.59		7790.395		700	3155.44	6236.62		4696.03
701	16978.6	7151.62		12065.11		701	4445.96	7467.54		5956.75
702	24740.6	10183.72		17462.16		702	5999.21	8493.54		7246.375
703	39546.5	15276.09		27411.3		703	8019.27	10397.41		9208.34
704	53072.9	21977.16		37525.03		704	9855.68	11693.23		10774.46
705	67145.8	27443.47		47294.64		705	12286.41	16493.47		14389.94
706	88253.5	33278.83		60766.17		706	15678.39	19490.13		17584.26
707	108319	43905.29		76112.15		707	17802.55	23239.97		20521.26
708	136861.7	52120.22		94490.96		708	22428.84	27803.19		25116.02

709	147657.7	63352.93		105505.3		709	26102.56	31758.7		28930.63
710	184453.5	73481.73		128967.6		710	30280.13	35404.24		32842.19
711	205245.1	83710.03		144477.6		711	34159.37	40975.73		37567.55
712	237610.3	97719.93		167665.1		712	39306.63	46091.95		42699.29
713	252921.1	106464.1		179692.6		713	41001.2	49696.49		45348.85
714	278372.7	113538.3		195955.5		714	45233.81	53593.58		49413.7
715	297922.7	116937.8		207430.3		715	48134.98	56651.95		52393.47
716	308019	128137.9		218078.5		716	50502.78	58443.47		54473.13
717	311418	138060.4		224739.2		717	53685.87	60654.93		57170.4
718	334017.2	139313.5		236665.4		718	53808.58	67952.75		60880.67
719	357675.4	142983.8		250329.6		719	54730.94	68148.35		61439.65
720	358903.9	146960.1		252932		720	57845.01	70270.52		64057.77
721	372547.1	145435.6		258991.4		721	59792.59	72704.64		66248.62
722	362941.2	146982.9		254962.1		722	55833.44	71665.23		63749.34
723	347782.2	144673.4		246227.8		723	54233.55	68204.74		61219.15
724	339592.1	138871.9		239232		724	52261.33	65116		58688.67
725	342368.4	133887.3		238127.9		725	51883.91	62878.55		57381.23
726	318973.6	132463.6		225718.6		726	49092.33	59948.27		54520.3
727	298445	129016.8		213730.9		727	46155.48	56284.8		51220.14
728	299440	122580.2		211010.1		728	44392.91	52771.72		48582.32
729	285947.1	120405.5		203176.3		729	43461.3	49196.62		46328.96
730	271186.9	111782.7		191484.8		730	37802.14	46183.76		41992.95
731	247059.6	106099.5		176579.6		731	37218.19	43536.18		40377.19
732	225188.8	98164.13		161676.5		732	35807.94	39314.49		37561.22
733	200312.2	91281.93		145797.1		733	31374.68	35680.68		33527.68
734	175778.7	80954.43		128366.6		734	27883.61	31280.05		29581.83
735	164667.8	78793.73		121730.8		735	26881.79	28757.06		27819.43
736	157336.7	70744.33		114040.5		736	23030.11	24354.83		23692.47
737	142619	63258.63		102938.8		737	20360.79	20551.66		20456.23
738	132836.1	57236.83		95036.47		738	16828.27	18567.52		17697.9
739	111306.8	52171.97		81739.39		739	14731.1	18509.11		16620.11
740	103286.2	45249.48		74267.84		740	13220.03	16494.18		14857.11

Table S16. Figure 5C data

C-1004						PCC7806				
	Yaxis move						Yaxis move			
	I	II	III	Average			I	II	III	Average
670	129398.4	357098.8	1090584	525693.8		670	1599941	42733.31	1018498	887057.6
671	235724.5	332204.8	1046844	538257.8		671	1622473	34848.97	907113.4	854811.9
672	159581.4	442726.7	843941.2	482083.1		672	1498860	30976.31	848058.9	792631.7
673	219440.5	505609.9	900130.5	541726.9		673	1465153	24763.33	679487.1	723134.5
674	343109.1	588362.8	1209206	713559.4		674	1464179	23310.09	798264.1	761917.7
675	443268.7	909389.2	1573885	975514.3		675	1305549	23634.83	903305.1	744163.1
676	577719.8	983963.7	1617572	1059752		676	1453376	22446.16	933648.4	803156.9
677	1255659	1374492	1835570	1488574		677	1560775	25139.42	947859.1	844591.3
678	1639469	1825979	2270345	1911931		678	1617676	23786.04	1132918	924793.5
679	1952911	2072161	2448753	2157941		679	1894495	24639.59	1191374	1036836
680	2091878	2254819	2265652	2204116		680	1649922	24017.68	1299892	991277.5
681	2346283	2376821	1829757	2184287		681	1729456	20465.67	1117481	955800.7
682	2286959	2400848	1851261	2179689		682	1525257	20965.12	1033033	859751.5
683	2281945	2479491	1816907	2192781		683	1377694	13920.54	882861.5	758158.5
684	2308628	2303272	1721610	2111170		684	1107698	12006.47	862386.3	660697
685	2186911	1790417	1538614	1838648		685	1127597	9231.986	805246.1	647358.3
686	1854655	1834577	1390762	1693331		686	828525	8900.281	587473	474966.1
687	1790232	1663596	1058353	1504060		687	793385.1	4378.509	275915.9	357893.2
688	1495558	1450414	1023449	1323140		688	841141.3	3038.066	205228.6	349802.7
689	1434731	1333556	710139.8	1159475		689	582340.2	5704.327	254566.2	280870.2
690	1251047	1021592	127660.8	800099.6		690	477699.5	2599.523	295641.5	258646.8
691	1080635	1173009	85408.36	779684.1		691	268570.8	1788.123	336413.4	202257.4
692	1056037	1047428	8315.372	703926.9		692	277611.4	3442.979	212993.5	164682.6
693	1105627	733574	84627.38	641276.2		693	191750.8	0	136557.2	109436
694	965631.3	490347.9	148349.9	534776.4		694	254559.2	4769.109	57780.29	105702.9
695	699617.8	580061.8	0	426559.9		695	79343.44	4852.941	0	28065.46
696	634637.6	501421.9	177812.7	437957.4		696	0	8400.888	118789	42396.61
697	495912.1	300359.2	148117.6	314796.3		697	217602.3	8016.708	30397.06	85338.7
698	528629.9	316620.1	68395.04	304548.3		698	222856.9	15607.86	159542	132668.9
699	582209.1	269852.2	19216.21	290425.9		699	70411.36	13886.92	100150.9	61483.06
700	245840.4	222476.7	125523	197946.7		700	338637	19157.02	100098.1	152630.7
701	458656.5	180672.1	200978.8	280102.5		701	462640.3	22196.91	295020.8	259952.7
702	501272.2	99107.54	258158.3	286179.3		702	486749.4	22180.78	256828.8	255253
703	586694.4	0	256542.3	281078.9		703	544081.6	34326.73	396771.3	325059.9
704	649527.9	369165.5	391381	470024.8		704	594687.9	41762.89	354012.6	330154.5
705	803794.8	288412.4	342945.1	478384.1		705	901907.9	48119.33	431185.2	460404.1
706	887460.2	326462.3	446226.9	553383.1		706	1035431	51129.47	520266.1	535608.8
707	749754.9	372879.8	697685.4	606773.4		707	1227960	55502.79	586553.3	623338.8
708	989331.2	258717	988346.9	745465		708	1214313	62651.87	566373.7	614446
709	998596.7	369887.5	1096177	821553.8		709	1418943	65981.27	570485.5	685136.4

710	1118209	414589.2	1146712	893169.9		710	1591603	77265.65	608692.8	759187.1
711	1355695	614698.4	1155479	1041958		711	1703917	80551.93	906528.7	896999.3
712	1511448	593521.8	1328924	1144631		712	1656452	80645.01	851901.7	862999.6
713	1527072	690254.8	1302314	1173213		713	2058987	87347.85	959428.6	1035254
714	1551609	664890.9	1215181	1143894		714	2126376	94805.05	1080412	1100531
715	1644946	815174.2	1322480	1260867		715	1962963	101032.1	1249770	1104588
716	1678702	881073.5	1614482	1391419		716	2449261	105364.2	1189798	1248141
717	1764599	864025.3	1795226	1474617		717	2641003	107611.5	1311593	1353402
718	1935162	771695.1	1945543	1550800		718	2519472	113619.2	1371082	1334724
719	2316400	984933.2	1832988	1711440		719	2225640	119500.2	1455654	1266931
720	2080800	974097.3	1853364	1636087		720	2318786	127144.4	1572450	1339460
721	2208735	939453.3	2056245	1734811		721	2729912	134313.3	1619121	1494449
722	2334755	1191126	2021272	1849051		722	2716820	140398.6	1720343	1525854
723	2159687	1146953	1957894	1754845		723	2698507	144570.1	1843871	1562316
724	2407913	1196814	1835738	1813488		724	2739611	150712.2	1921203	1603842
725	2482890	1151007	2065402	1899766		725	2971393	156492.8	1879746	1669211
726	2452151	1208477	2016640	1892423		726	3161318	155900.8	1945610	1754276
727	2038318	1155955	1913290	1702521		727	3010723	154388	2302141	1822418
728	2199343	1030231	2044929	1758168		728	2896850	147597.5	2405396	1816614
729	1935887	1029521	1886094	1617167		729	2806269	143337.8	2297801	1749136
730	1483325	1019627	1886906	1463286		730	2805527	141550.7	2290018	1745699
731	1367820	753907	1650395	1257374		731	2528566	134279.2	2177620	1613488
732	1058429	914922.2	1357993	1110448		732	1943943	130620.8	2209886	1428150
733	714983.2	849635.1	1092217	885611.7		733	2021507	131086.2	1979972	1377522
734	762500.7	815062.5	901661.3	826408.2		734	1630711	122691.1	1639562	1130988
735	689810.7	631317.8	1054875	792001.3		735	1873391	115037.6	1863230	1283886
736	591618.7	723436.8	963390.5	759482		736	1439355	107855.2	1887986	1145065
737	493144.8	616435.8	685364.4	598315		737	1422878	105042.4	1620603	1049508
738	424628.4	480959.4	680090	528559.3		738	1465347	100087.5	1596574	1054003
739	43466.22	379012.4	593941.3	338806.7		739	1328100	98153.61	1608278	1011510
740	0	453984.9	551217.7	335067.5		740	855359.8	92486.49	1538397	828747.8

Table S17. Figure 5D Data

C-1004						PCC7806				
	Yaxis move						Yaxis move			
	I	II		Average			I	II		Average
670	135409.4	57545.63		96477.53		670	99122.86	38039.54		68581.2
671	156906.9	60323.87		108615.4		671	84981.5	35420.03		60200.77
672	161264.2	52836.03		107050.1		672	82940.15	37886.9		60413.53
673	174746.7	68078.97		121412.8		673	71334.9	35796.51		53565.71
674	221516.7	78767.64		150142.2		674	75842.45	34863.9		55353.18
675	263020.6	108540.4		185780.5		675	78840.43	38586.06		58713.25
676	309507.8	152201.9		230854.8		676	83005.28	38504.85		60755.06
677	433023	196592.1		314807.6		677	85630.31	39957.27		62793.79
678	511114	242875.5		376994.7		678	85908.69	41978.98		63943.83
679	614958.2	282677.6		448817.9		679	89692.36	45535.97		67614.17
680	611793.9	297088.7		454441.3		680	90071.84	44686.27		67379.06
681	657001.9	316886.2		486944.1		681	82361.77	39278.2		60819.99
682	637724.2	330988.4		484356.3		682	78626.66	36988.46		57807.56
683	535072	329907.6		432489.8		683	72534.22	31747.09		52140.65
684	511976.6	281772.9		396874.7		684	55416.24	24277.03		39846.63
685	492081.7	261464.6		376773.1		685	53408.34	27567.83		40488.08
686	444767.1	220357.7		332562.4		686	46127.23	18643.83		32385.53
687	362616.6	181110.3		271863.4		687	34542.8	12756.81		23649.81
688	305806	153315.1		229560.6		688	26705.4	9560.439		18132.92
689	261036.1	115086.6		188061.3		689	22162.14	3268.704		12715.42
690	188278.1	78017.44		133147.8		690	18523	594.2378		9558.618
691	159683.2	59423.25		109553.2		691	12712.48	0		6356.238
692	137160.4	33546.84		85353.6		692	10795.43	2235.665		6515.549
693	132381.3	18526.33		75453.82		693	9004.037	1582.375		5293.206
694	69411.61	16938.18		43174.9		694	2808.636	6680.203		4744.42
695	70030.38	13992.3		42011.34		695	0	3764.248		1882.124
696	44904.92	15100.75		30002.84		696	7346.505	4273.873		5810.189
697	31791.46	11463.66		21627.56		697	9974.016	7549.076		8761.546
698	61532.96	0		30766.48		698	17178.88	10852.67		14015.77
699	77919.04	7946.713		42932.88		699	19568.49	14706.15		17137.32
700	96774.93	7424.177		52099.55		700	21820.38	13755.94		17788.16
701	120386.5	13866.35		67126.44		701	27655.13	24566.11		26110.62
702	98724.31	26602.49		62663.4		702	32138.31	26235.3		29186.8
703	128786.4	38803.84		83795.12		703	35661.7	30918.33		33290.02
704	159914.1	49547.93		104731		704	49515.87	41351.1		45433.48
705	179849.9	63718.08		121784		705	57601.95	44732.32		51167.14
706	234702.7	86785.89		160744.3		706	56538.79	47902.04		52220.41
707	248287.3	91624.78		169956.1		707	59439.5	55691.31		57565.4
708	217727.8	107855.7		162791.8		708	73280.56	56382.04		64831.3
709	227364.4	112649.6		170007		709	79684.49	60169.69		69927.09

710	236421.1	113607.6		175014.4		710	79354.04	74413.94		76883.99
711	220821.4	120451.6		170636.5		711	82175.5	73902.1		78038.8
712	224315.5	128462.2		176388.8		712	88527.23	74941.99		81734.61
713	234932.4	135857.6		185395		713	103185.2	76866.22		90025.73
714	281810	154161.9		217985.9		714	103270.8	82489.26		92880.02
715	301859.6	157261.9		229560.7		715	108519	86593.47		97556.23
716	295830	177045.1		236437.5		716	110449.7	88400.58		99425.14
717	326285.7	176800.4		251543		717	113917.2	95077.21		104497.2
718	301046.4	182452.4		241749.4		718	119581.8	99694.32		109638.1
719	262377.2	183759		223068.1		719	118997.9	108210.6		113604.3
720	252866.9	199260.2		226063.5		720	120081.6	108185.2		114133.4
721	239385.4	203257.3		221321.4		721	130233	112926.9		121580
722	290010.8	236247		263128.9		722	126605.4	123292.3		124948.9
723	309904.6	227419.4		268662		723	139014	127297.2		133155.6
724	325980.8	223460.6		274720.7		724	137660.3	127985.1		132822.7
725	334285.8	244284		289284.9		725	135522.6	126074.7		130798.7
726	332840.3	257244.7		295042.5		726	137346.7	128643		132994.9
727	263485.1	240528		252006.6		727	137766.5	127691.1		132728.8
728	240224.1	201015.4		220619.8		728	127798	112495.5		120146.8
729	222698.7	200833.9		211766.3		729	118622.7	108233.3		113428
730	206429.3	187736.9		197083.1		730	116290.6	107472.4		111881.5
731	175932.5	185082.7		180507.6		731	101218.6	105943.8		103581.2
732	146710.8	172499.5		159605.2		732	88754.55	104733.7		96744.14
733	106766.4	150270.4		128518.4		733	83261	94112.2		88686.6
734	88722.92	131257		109989.9		734	73608.89	90192.23		81900.56
735	76578.81	113856.8		95217.82		735	67534.29	82575.12		75054.7
736	78676.78	92981.46		85829.12		736	62862.14	75298.56		69080.35
737	67073.51	78240.45		72656.98		737	53696.84	69023.65		61360.25
738	35131.81	61542.62		48337.22		738	39829.09	70897.3		55363.19
739	6731.962	50832.15		28782.06		739	32388.68	64746.69		48567.69
740	8402.404	53638.25		31020.33		740	29231.2	61218.73		45224.97

Table S18. Figure 6A Data.

Strains	Temperature	Values
PCC7806	20	9.422636
PCC7806	20	15.77546
PCC7806	20	23.06126
C1004	20	12.12951
C1004	20	11.48456
C1004	20	11.8072
PCC7806	40	2.451731
PCC7806	40	5.677112
PCC7806	40	6.533976
C1004	40	2.205661
C1004	40	2.440294
C1004	40	2.732079

Table S19. Figure 6B Data.

Strain	Temperature	Value
PCC7806	20	22.05201
PCC7806	20	32.22926
PCC7806	20	52.85678
PCC7806	40	115.2062
PCC7806	40	55.28829
PCC7806	40	76.94443
C1004	20	7.46263
C1004	20	16.05605
C1004	20	9.43463
C1004	40	21.50544
C1004	40	15.48302
C1004	40	25.37272

Table S20. Figure 6C Data.

Strain	Temperature	Value
PCC7806	20	35.25623
PCC7806	20	31.2286
PCC7806	20	34.53517
PCC7806	40	3.458581
PCC7806	40	7.37234
PCC7806	40	4.589404
C1004	20	46.2
C1004	20	52.26244
C1004	20	50.62089
C1004	40	8.161
C1004	40	10.6466

C1004	40	24.56881

Table S21. Table 1 Data.

**Temperature**	**Strain**	**Value**
P700		
20	PCC7806	38.7
20	PCC7806	22.85
20	PCC7806	32.85
20	C1004	54.58
20	C1004	50
20	C1004	45.73
40	PCC7806	58.57
40	PCC7806	98.57
40	PCC7806	93.7
40	C1004	43.64
40	C1004	89.42
40	C1004	87.142
B6f		
20	PCC7806	20
20	PCC7806	46.5
20	PCC7806	35.22
20	C1004	15
20	C1004	24.2
20	C1004	49.38
40	PCC7806	446.6
40	PCC7806	666.6
40	PCC7806	708.8
40	C1004	1161
40	C1004	1722
40	C1004	600
PC		
20	PCC7806	54.14
20	PCC7806	124.25
20	PCC7806	57.23
20	C1004	66.59
20	C1004	180.42
20	C1004	60.212
40	PCC7806	2142
40	PCC7806	842.5
40	PCC7806	925.5
40	C1004	98.6
40	C1004	126.97
40	C1004	155.34

Table S22. Figure 8 Data

Species	Temperature	Values
PCC7806	20	7.2
PCC7806	20	12.33075
PCC7806	20	19.15931
PCC7806	40	36
PCC7806	40	32
PCC7806	40	34
C1004	20	44.1383
C1004	20	54
C1004	20	33.54977
C1004	40	18
C1004	40	20.88
C1004	40	19.44

Table S23. Figure 9 Data.

Heat Shock			
		Fold_Change_mean	Fold_Change_SE
groEL	1004	1.585032951	0.051328
groEL	7806	1.061168537	0.281544
groES	1004	3.038096851	0.894719
groES	7806	0.844364201	0.029819
clpB1	1004	2.227613636	0.301228
clpB1	7806	1.305239381	0.171018
dnaK1	1004	1.478665025	0.072325
dnaK1	7806	1.337829336	0.097867
Photosynthesis			
		Fold_Change_mean	Fold_Change_SE
psbA	1004	27.48204417	13.14148
psbA	7806	1.342543073	0.360922
petE	1004	0.958489593	0.108318
petE	7806	0.309782584	0.071264
petJ	1004	5.9	0.34
petJ	7806	3.71	0.08
sod	1004	1.215952237	0.414888
sod	7806	0.449570597	0.041065
Krebs			
		Fold_Change_mean	Fold_Change_SE

sdh	1004	7.415596087	2.295462
	7806	-5.173384939	1.693151
icdh	1004	1.882752081	0.376835
	7806	1.162030209	0.521424
